# Bread Consumption and Cancer Risk: Systematic Review and Meta-Analysis of Prospective Cohort Studies

**DOI:** 10.1016/j.cdnut.2024.104501

**Published:** 2024-11-02

**Authors:** Glenn A Gaesser, Siddhartha S Angadi, Craig Paterson, Julie Miller Jones

**Affiliations:** 1 College of Health Solutions, Arizona State University, Phoenix, AZ, United States; 2 Department of Kinesiology, University of Virginia, Charlottesville, VA, United States; 3 Population Health Sciences, Bristol Medical School, University of Bristol, United Kingdom; 4 Department of Family, Consumer, and Nutritional Science, St. Catherine University, Minneapolis, MN, United States

**Keywords:** diet, whole-grain, refined grain, grains, grain foods, baked goods, acrylamide

## Abstract

Because bread can contain potential carcinogens such as acrylamide, and is widely consumed, we conducted a systematic review and meta-analysis to determine whether bread consumption is associated with increased cancer risk. PubMed and Medline databases were searched up to 1 March 2024, for studies that provided hazard ratios (HRs) (or similar) for bread consumption and cancer incidence or mortality. Only prospective cohort studies were included. We used the Preferred Reporting Items of Systematic reviews and Meta-Analyses checklist. Meta-analysis was performed with Cochrane’s RevMan 5.4.1 software using a DerSimonian–Laird random-effects model. Heterogeneity was assessed with Cochrane’s *Q* (χ^2^) and *I*^2^ statistics, and publication bias was assessed with Egger’s test. Twenty-four publications met inclusion criteria, including 1,887,074 adults, and were included in the systematic review. Ten publications that provided HRs were included in the meta-analysis for highest compared with lowest intakes, and an additional 7 publications that provided mortality or incident rate ratios or relative risks were included in supplemental meta-analyses. Of 108 reported HRs (or similar), 97 (79%) were either not statistically significant (*n* = 86) or indicated lower cancer risk (*n* = 11) associated with the highest intakes of bread. The meta-analysis indicated that bread intake was not associated with site-specific cancer risk [HR: 1.01; 95% confidence interval (CI): 0.89, 1.14; *P* = 0.92; 8 publications] or total cancer mortality (HR: 0.90; 95% CI: 0.73, 1.11; *P* = 0.32; 2 publications). Supplemental meta-analyses using all risk estimates in addition to HRs confirmed these findings. Whole-grain bread was associated with a lower site-specific cancer risk, mainly because of reduced colorectal cancer risk. Results of the systematic review and meta-analysis indicate that bread consumption is not associated with increased site-specific cancer risk, whereas high whole-grain/nonwhite bread consumption is associated with lower total cancer mortality and colorectal cancer risk.

This study was registered at Clinical Trials Registry of PROSPERO as registration number CRD42023414156.

## Introduction

Bread is one of the most widely consumed foods in the world. As a major grain food, bread is an important nutrient-dense food that contributes shortfall nutrients [1,2]. However, bread can be a significant source of potentially harmful compounds formed during processing, such as acrylamide, heterocyclic amines, and polycyclic aromatic hydrocarbons [[3], [4], [5], [6]]. These compounds are formed in any carbohydrate food during dry heating to temperatures >120°C, as occurs in frying and baking, and are among many components associated with Maillard browning, which is crucial for color and flavor in bread. Acrylamide, for example, has been shown to cause cancer in animals exposed to very high doses, and in 1994, the International Agency for Research on Cancer (IARC) listed acrylamide as “probably carcinogenic to humans” [7]. Subsequently, the United States National Toxicology Program (NTP) classified acrylamide as “reasonably anticipated to be a human carcinogen” [8]. As a source of acrylamide, bread is cited as a reason to avoid ultraprocessed foods such as bread [9,10].

However, since the initial IARC statement 30 y ago, results from epidemiologic studies assessing the association between dietary acrylamide exposure and cancer risk have been inconclusive [[11], [12], [13], [14], [15], [16], [17]]. Despite the inconsistent findings from epidemiologic studies on the association between acrylamide exposure from foods and cancer in humans, both the United States NTP and the Joint FAO/WHO Expert Committee on Food Additives consider acrylamide to be a concern to human health [18].

Bread could also conceivably increase cancer risk via glycemic index (GI). Many breads have a moderate to high GI [19]. Several meta-analyses have demonstrated that a high dietary GI is associated with increased cancer risk [[20], [21], [22], [23], [24]], although the risk estimates are generally low, and not all meta-analyses have demonstrated higher cancer risk associated with high dietary GI [[25], [26], [27]].

Results from 3 meta-analyses indicated that bread consumption was associated with a lower risk of cancer mortality [[28], [29], [30]]. All 3 of these meta-analyses were published in 2016 and included only 2–4 publications in their meta-analyses on bread consumption. Moreover, only whole-grain or nonwhite bread was examined. To our knowledge, no systematic review or meta-analysis has examined the association between bread intake and site-specific cancer risk, or whether cancer risk differs by bread type.

Thus, this systematic review and meta-analysis aimed to determine whether high intake of bread is associated with total and site-specific cancer risk. Accordingly, for the meta-analysis, we focused specifically on categorical analysis of highest compared with lowest intakes in prospective cohort studies.

## Methods

The PROSPERO registration number for this study is CRD42023414156. The PRISMA checklist was used for this study [31,32].

### Search strategy

PubMed and Medline databases were searched and screened independently by 2 of the authors (GAG and SSA) to identify relevant prospective cohort studies that published cancer incidence or mortality data in relation to bread intake. The search strategy included “bread AND cancer.” Any disagreements in studies selected by the 2 authors were resolved in consultation with a third author (CP). Databases were searched from inception to 1 March 2024. Reference lists and electronic citation records of all retrieved articles, including those from previously published meta-analyses, were also reviewed for additional studies not identified in the initial search. Potentially relevant, previously published meta-analyses were identified with a “grains AND cancer” search of PubMed and Medline databases.

### Study selection

Only full-text English language publications that reported data from an original, peer-reviewed prospective cohort study of adults were included. Each publication included in the systematic review had to provide an incidence rate ratio (IRR) or mortality rate ratio (MRR), hazard ratio (HR), relative risk (RR), or odds ratio (OR) with 95% CIs. Only studies that analyzed bread as a distinct food were included in this review. Publications that included bread as part of a grains food group or dietary pattern were excluded. Case-control studies, reviews, conference proceedings, and abstracts were also excluded.

### Data extraction and quality assessment

For each included study, the following information was extracted: name of first author; year of publication; name of cohort, sex, age range, and total number of participants; mean/median duration (or range of years) of follow-up; method of dietary assessment; bread type analyzed and quantity consumed; adjustments in the statistical model; and cancer outcomes (site, incidence, mortality, and total number of cases). The quantification of bread consumption in highest and lowest intake groups, as well as in dose-response studies, was also recorded. For studies that reported >1 HR for a specific cancer outcome, we used the HR in the most fully adjusted statistical model.

Study quality was assessed independently by 2 investigators (GAG and SSA) with the Newcastle-Ottawa scale [33], with consultation from a third reviewer in the event of disagreement. The scale includes 3 domains to assess study quality: selection (4 points), comparability (2 points), and outcome (3 points). Studies scoring 7–9, 4–6, and 0–3 points were rated as high-quality, moderate-quality, and low-quality studies, respectively.

### Data synthesis

Meta-analysis was performed using Cochrane RevMan 5.4.1 software. Data were pooled using a DerSimonian–Laird random-effects model to account for potential heterogeneity within and between studies. Aggregation of the data and analysis was performed by 1 author (GAG). Because our intent was to determine whether high intakes of bread were associated with increased cancer risk, only studies that compared highest with lowest intakes of bread were subjected to meta-analysis. Also, because IRRs, MRRs, HRs, RRs, and ORs are calculated differently and should not be used interchangeably [34], we restricted our primary meta-analysis on highest compared with lowest intakes to only those studies that reported HRs because these were the most frequently reported outcome. These results represent the primary focus of this review and are reported as HR with corresponding 95% CIs. However, in the interest of providing a comprehensive examination of the entirety of evidence, and in line with previous reviews in this area [[28], [29], [30]], we conducted additional, expanded analyses that included all reported outcome measures from each publication. The results of these expanded analyses are reported as μ with corresponding 95% CIs as they represent a summary effect taken from different outcome measures. We note that the interpretation of these models should be made with caution as they combine related but different statistical outcomes.

Additional sensitivity analyses were performed by systematically removing 1 study at a time and recalculating the summary association to determine the robustness of the results and the impact of single study on the HR (μ) and heterogeneity. To further interrogate the potentially differing effects of bread type on the observed outcome, subgroup analysis by bread type was also conducted.

Heterogeneity was assessed with both χ^2^ (*P* < 0.05) and *I*^2^ statistics. For *I*^2^, values of 0%–25%, 25%–50%, 50%–75%, and 75%–100% were considered modest, modest-to-moderate, moderate-to-strong, and strong heterogeneity, respectively [35]. Publication bias was assessed with Egger’s regression symmetry test for meta-analyses that included ≥6 studies, with significant bias established at *P* < 0.10 [36]. Funnel plots were generated only when the meta-analysis included ≥10 studies [35].

## Results

**FIGURE 1:**
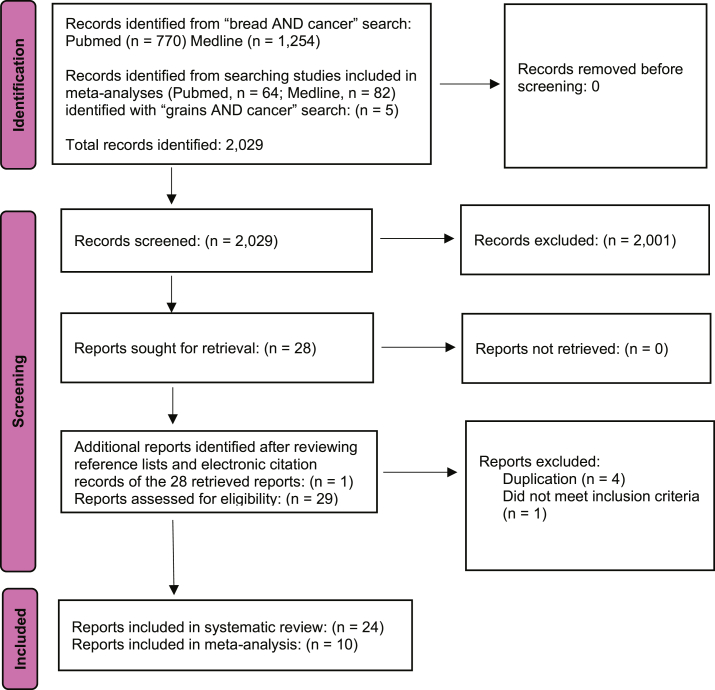
PRISMA flow diagram for study selection.

The flowchart for publication selection is presented in Figure 1 [32]. A total of 770 records were identified on PubMed and 1254 on Medline in our “bread AND cancer” search. Our “grains AND cancer” meta-analysis search produced 64 records on PubMed and 82 records on Medline. Three of these records included meta-analyses of bread consumption and cancer mortality, which together included a total of 5 cohort studies. Thus, 2029 records were screened. After title/abstract screening and identification of case-control and dietary pattern studies, 2001 records were excluded, leaving 28 full-text articles that were assessed for eligibility. After reviewing reference lists and electronic citation records of each of these 28 publications, 1 additional publication was identified for eligibility assessment. Thus, 29 publications were assessed for eligibility. Four publications were excluded as duplications, and 1 additional publication did not meet inclusion criteria, leaving a total of 24 publications that met inclusion criteria.

Of the 24 publications that met inclusion criteria, 10 provided HRs and corresponding 95% CIs for cancer mortality or incidence when comparing highest with lowest intakes of bread [[37], [38], [39], [40], [41], [42], [43], [44], [45], [46]] (Table 1) and were included in our meta-analysis. Seven additional publications provided either MRR [47,48], IRR [[49], [50], [51]], or RR [52,53], and were included in the supplemental meta-analysis (Table 1). Seven studies provided results only for dose-response analyses [[54], [55], [56], [57], [58], [59], [60]] (Table 2) and were included in the systematic review.

**TABLE 1:** Associations between bread intake (highest vs. lowest) and cancer risk in prospective cohort studies.

Study	Outcome	Comparison	Bread type	HR/HRR/RR/RRR/MRR/IRR (95% CI)	
Total cancer					
Appleby et al. [47], 2002 Health Food Shoppers study	Mortality	“Daily” vs. “Less than daily”	Wholemeal	MRR	1.01 (0.85, 1.20)^1^
Cordova et al. [39], 2023 European Prospective Investigation into Cancer and Nutrition	Mortality	“Daily” vs. “Less than Weekly”	Whole grain	HR	**0.98 (0.86, 1.12)**^2^
Jacobs et al. [41], 2001 National Health Screening Service of Norway	Mortality	Whole-grain bread score: 2.25–5.4 (∼9 slices/d) vs. ≤0.60 (∼1 slice/d)	Whole grain	HRR	**0.79 (0.62, 1.02)**^2^
Johnsen et al. [48], 2015 Scandinavian HELGA cohort	Mortality (Males)	Median intake of Q4 (194 g/d) vs. Q1 (13 g/d)	Nonwhite	MRR	0.79 (0.64, 0.97)^1^
	Mortality (Females)	Median intake of Q4 (180 g/d) vs. Q1 (23 g/d)	Nonwhite		0.89 (0.75, 1.05)^1^
	Mortality (Males)	Median intake of Q4 (38 g/d) vs. Q1 (1 g/d)	Crisp		0.83 (0.67, 1.03)^1^
	Mortality (Females)	Median intake of Q4 (31 g/d) vs. Q1 (0.6 g/d)	Crisp		0.87 (0.74, 1.02)^1^
Colorectal cancer					
Abe et al. [37], 2014 Japan Public Health Center-based prospective study	Incidence (Males)	Q4 (45–720 g/d) vs Q1 (0–4 g/d)	Total bread	HR	**0.98 (0.78, 1.23)**^2^
	Incidence (Females)	Q4 (47–720 g/d) vs Q1 (0–4 g/d)	Total bread		**1.01 (0.75, 1.36)**^2^
Appleby et al. [47], 2002 Health Food Shoppers study	Mortality	“Daily” vs. “Less than daily”	Wholemeal	MRR	1.21 (0.76, 1.93)^1^
Bakken et al. [38], 2016 Norwegian Women and Cancer Study	Incidence	High (>180 g/d) vs. Zero/seldom (0 g/d)	Whole grain – Total colorectal	HR	**0.85 (0.66, 1.09)**^2^
			Whole grain - colon		0.88 (0.66, 1.18)
			Whole grain – proximal colon		0.66 (0.44, 0.98)
			Whole grain – distal colon		1.14 (0.69, 1.88)
			Whole grain – rectal		0.78 (0.47, 1.27)
Jin et al. [42], 2023 UK Biobank	Incidence	Tertile 3 vs. Tertile 1	White – total colorectal	HR	**1.22 (1.08, 1.37)**^2^
			White – colon		1.22 (1.05, 1.43)
			White – rectal		1.35 (1.06, 1.70)
Kyro et al. [50], 2013 Scandinavian HELGA cohort	Incidence	Q4 [>153 g/d (males), >163 g/d (females)] vs. Q1 [≤26 g/d (males), ≤63 g/d (females)]	Whole grain	IRR	0.81 (0.64, 1.02)^1^
	Incidence	Q4 [>30 g/d (males), >11 g/d (females)] vs. Q1 [≤2 g/d (males), ≤1 g/d (females)]	Crisp		0.81 (0.64,1.02)^1^
Larsson et al. [51], 2005 Swedish Mammography Cohort	Incidence	[≥2 slices/d vs. <4 slices/wk]	Whole-grain rye	IRR	0.74 (0.55, 0.98)^1^
Sanjoaquin et al. [53], 2004 Oxford Vegetarian Study	Incidence	[15+ slices/wk vs. <15 slices/wk]	Brown	RR	0.90 (0.59, 1.38)^1^
			White		2.11 (1.17, 3.81)^1^
Breast cancer					
Appleby et al. [47], 2002 Health Food Shoppers study	Mortality	“Daily” vs. “Less than daily”	Wholemeal	MRR	1.22 (0.75, 1.97)^1^
Haraldsdottir et al. [40], 2018 Age Gene Environment Susceptibility (AGES) -Reykjavik cohort	Incidence	Daily or more vs. Less than daily	Rye	HR	**1.80 (1.10, 2.90)**^2^
			Whole wheat		**0.80 (0.50, 1.30)**^2^
Sonestedt et al. [45], 2008 Malmo Diet and Cancer cohort	Incidence	Q5 vs. Q1 Median intake: High-fiber bread: 65 g/d vs. 0 g/d Low-fiber bread: 116 g/d vs. 18 g/d	High-fiber bread	HR	**0.75 (0.57, 0.98)**^2^
			Low-fiber bread		**1.18 (0.89, 1.55)**^2^
Prostate cancer					
Appleby et al. [47], 2002 Health Food Shoppers study	Mortality	“Daily” vs. “Less than daily”	Wholemeal	MRR	1.24 (0.59, 2.57)^1^
Egeberg et al. [49], 2011 Diet, Cancer and Health study Denmark	Incidence	Q3 vs. Q1: Whole grain: >40 g/d vs. ≤17 g/d Whole-grain rye: >113 g/d vs. ≤63 g/d	Whole grain	IRR	1.07 (0.90, 1.28)^1^
			Whole-grain rye		0.89 (0.73, 1.08)^1^
Lan et al. [43], 2021 NIH-AARP Diet and Health Study	Incidence and Mortality	Dark bread: ≥3 times/wk vs. <11 times/y White bread: 2 times/d vs. ≤2 times/wk	Dark (Nonadvanced)	HR	1.06 (1.01, 1.11)
			Dark (Advanced)		0.95 (0.84, 1.07)
			Dark (Mortality)		**0.92 (0.75, 1.13)**^2^
			White (Nonadvanced)		0.95 (0.91, 1.02)
			White (Advanced)		1.12 (0.96, 1.30)
			White (Mortality)		**1.20 (0.92, 1.57)**^2^
Torfadottir et al. [46], 2012 Age Gene Environment Susceptibility (AGES) – Reykjavik cohort	Incidence	Daily vs. Less than daily	Rye (Total)	HR	**0.69 (0.45, 1.06)**^2^
			Rye (Advanced)		0.64 (0.25, 1.61)
			Whole wheat (Total)		**1.07 (0.75, 1.53)**^2^
			Whole wheat (Advanced)		0.96 (0.43, 2.14)
Esophageal cancer					
Skeie et al. [44], 2016 HELGA cohort	Incidence	Tertile 3 vs. 1 Males: 129.6–520 g/d vs. 0–59.6 g/d Females: 113.8–520 g/d vs. 0–65.8 g/d	Whole grain	HR	**0.63 (0.35, 1.14)**^2^
	Incidence	Tertile 3 vs. 1 Males: 13.0–280.1 g/d vs. 0–2.1 g/d Females: 10.5–184.6 g/d vs. 0–2.0 g/d	Crisp		**1.47 (0.84, 2.56)**^2^
Ovarian cancer					
Hedelin et al. [52], 2011 Scandinavian Women’s Lifestyle and Health Cohort	Incidence	Quartile 4 vs. Quartile 1	Whole grain	RR	1.48 (0.95, 2.31)^1^
Stomach cancer					
Appleby 2002 [47] Health Food Shoppers study	Mortality	“Daily” vs. “Less than daily”	Wholemeal	MRR	0.84 (0.42, 1.67)^1^
Pancreatic cancer					
Appleby 2002 [47] Health Food Shoppers study	Mortality	“Daily” vs. “Less than daily”	Wholemeal	MRR	0.86 (0.43, 1.73)^1^
Lung cancer					
Appleby et al. [47], 2002 Health Food Shoppers study	Mortality	“Daily” vs. “less than daily”	Wholemeal	MRR	1.08 (0.67, 1.76)^1^

Abbreviations: CI, confidence interval; HR, hazard ratio; HRR, hazard rate ratio; IRR, incidence rate ratio: MRR, mortality rate ratio; RR, relative risk.

1 included in supplemental meta-analysis.

2 Included in primary meta-analysis restricted to HRs

**TABLE 2:** Associations between bread intake (dose-response) and cancer risk in prospective cohort studies.

Study	Outcome	Bread type (dose)	HR/HRR/RR/RRR/MRR/IRR (95% CI)	
Total cancer				
Johnsen et al. [48], 2015 Scandinavian HELGA cohort	Mortality (males)	Nonwhite (per doubling)	MRR	0.96 (0.92, 0.99)
	Mortality (females)	Nonwhite (per doubling)		0.97 (0.94, 1.00)
	Mortality (males)	Crisp (per doubling)		0.96 (0.93, 1.00)
	Mortality (females)	Crisp (per doubling)		0.98 (0.95, 1.00)
Von Ruesten et al. [60], 2013 EPIC-Potsdam	Incidence	Whole grain (per 50 g/d)	HR	0.94 (0.84, 1.05)
		Other bread (per 50 g/d)		0.98 (0.88, 1.09)
Colorectal cancer				
Egeberg et al. [56], 2010 Diet, Cancer and Health study Denmark	Incidence (males)	Whole grain (per 25 g) – colon	IRR	0.89 (0.82, 0.97)
		Whole-grain rye (per 25 g) - colon		0.94 (0.88, 1.01)
		Whole grain (per 25g) – rectal		0.95 (0.86, 1.04)
		Whole-grain rye (per 25 g) - rectal		0.97 (0.89, 1.05)
	Incidence (females)	Whole grain (per 25 g) – colon		0.97 (0.89, 1.06)
		Whole-grain rye (per 25 g) – colon		1.01 (0.92, 1.11)
		Whole grain (per 25 g) – rectal		1.05 (0.94, 1.16)
		Whole-grain rye (per 25 g) – rectal		1.04 (0.92, 1.18)
Papadimitriou et al. [58], 2023 European Prospective Investigation into Cancer and Nutrition	Incidence	Nonwhite (per SD)	HR	0.93 (0.90, 0.97)
		Nonwhite (per SD) – males		0.89 (0.84, 0.94)
		Nonwhite (per SD) – females		0.99 (0.94, 1.05)
		White (per SD)		1.05 (1.01, 1.09)
		Bread (per SD)		0.98 (0.94, 1.01)
		Nonwhite (per SD) – colon		0.93 (0.88, 0.98)
		Nonwhite (per SD) – rectum		0.95 (0.89, 1.01)
		Nonwhite (per SD) – proximal		0.95 (0.88, 1.02)
		Nonwhite (per SD) – distal		0.93 (0.86, 1.00)
Papadimitriou et al. [58], 2023 Netherlands Cohort Study	Incidence	Nonwhite (per SD)	HR	1.00 (0.95, 1.05)
		Nonwhite (per SD) – colon		0.98 (0.93, 1.04)
		Nonwhite (per SD) – rectum		1.06 (0.98, 1.14)
		Nonwhite (per SD) – proximal		0.97 (0.91, 1.05)
		Nonwhite (per SD) – distal		1.00 (0.93, 1.07)
		Nonwhite (per SD) – males		1.00 (0.95, 1.07)
		Nonwhite (per SD) – females		1.00 (0.91, 1.11)
Kyro et al. [50], 2013 Scandinavian HELGA cohort	Incidence	Whole grain (per 25 g)	IRR	0.96 (0.93, 0.99)
	Incidence	Crisp (per 25 g)		0.96 (0.86, 1.08)
Larsson et al. [51], 2005 Swedish Mammography Cohort	Incidence	Whole-grain rye (per slice/d)	IRR	0.88 (0.78, 0.99)
Jin et al. [42], 2023 UK Biobank	Incidence	White (per SD)	HR	1.10 (1.05, 1.16)
		White (per SD) – colon		1.09 (1.02, 1.16)
		White (per SD) – rectal		1.15 (1.01, 1.25)
		Wholemeal (per SD) – colon		0.94 (0.87, 1.00)
		Wholemeal (per SD) – rectal		0.96 (0.87, 1.07)
Breast cancer				
Andersen et al. [55], 2020 Danish – Diet, Cancer and Health cohort	Mortality	Whole grain (per 40 g)	HR	1.04 (0.94, 1.15)
		Rye (per 50 g)		1.11 (0.96, 1.29)
Farvid et al. [57], 2016 Nurses’ Health Study II	Incidence	Premenopausal intake		
		Dark (per 2 servings/wk) – all cases	RR	0.99 (0.98, 1.01)
		White (per 2 servings/wk) – all cases		1.02 (1.01, 1.04)
		Dark (per 2 servings/wk) –premenopausal cases		0.98 (0.96, 1.01)
		White (per 2 servings/wk) – premenopausal cases		1.03 (1.00, 1.05)
		Dark (per 2 servings/wk) – postmenopausal cases		1.01 (0.99, 1.04)
		White (per 2 servings/wk) – postmenopausal cases		1.03 (1.00, 1.06)
		Adolescent intake		
		Dark (per 2 servings/wk) – all cases	RR	0.99 (0.96, 1.02)
		White (per 2 servings/wk) – all cases		1.01 (0.99, 1.02)
		Dark (per 2 servings/wk) – premenopausal cases		0.98 (0.94, 1.03)
		White (per 2 servings/wk) – premenopausal cases		1.01 (0.99, 1.03)
		Dark (per 2 servings/wk) – postmenopausal cases		1.02 (0.97, 1.06)
		White (per 2 servings/wk) – postmenopausal cases		1.00 (0.98, 1.02)
Prostate cancer				
Egeberg et al. [49], 2011 Diet, Cancer and Health study Denmark	Incidence	Whole grain (per 25 g)	IRR	1.01 (0.98, 1.04)
		Rye (per 25 g)		0.99 (0.96, 1.03)
Pancreatic cancer				
Schacht et al. [59], 2021 Danish, Diet, Cancer and Health Cohort	Incidence	Wholemeal (per 40 g/d)	HR	0.94 (0.86, 1.03)
		Rye (per 50 g/d)		0.89 (0.79, 1.00)
Endometrial cancer				
Aarestrup et al. [54], 2012 Diet, Cancer and Health study Denmark	Incidence	Rye (per 50 g)	IRR	0.86 (0.70, 1.06)
		Whole grain (per 50 g)		1.09 (0.93, 1.28)
		Crisp (per 5 g)		1.04 (0.97, 1.11)
Esophageal cancer				
Skeie et al. [44], 2016 HELGA cohort	Incidence	Whole grain (per 25 g/d)	HR	0.88 (0.80, 0.96)
	Incidence	Crisp (per 25 g/d)		0.1.06 (0.80, 1.41)

Abbreviations: CI, confidence interval; HR, hazard ratio; HRR, hazard rate ratio; IRR, incidence rate ratio; MRR, mortality rate ratio; RR, relative risk; SD, standard deviation.

Details of the 24 publications are presented in Supplemental Table 1. Of the 24 cohorts, 21 were from Europe, 2 from the United States, and 1 from Japan. The 24 studies included a total of 1,887,074 adults (63.2% females). Twenty-two of the 24 studies used some variation of the food-frequency questionnaire (FFQ) for dietary assessment.

Of the 21 publications from European cohorts, 13 were subpopulations within the larger European Investigation into Cancer and Nutrition (EPIC) cohort [38,39,44,45,[48], [49], [50],[54], [55], [56],[58], [59], [60]]. Two publications included data from 7–10 countries within the EPIC cohort, 1 of which examined colorectal incidence [58] and the other total cancer mortality [39]. Three publications were from the HELGA cohort [44,48,50], which is part of EPIC, and consists of the Norwegian Women and Cancer Study, the Danish Diet, Cancer and Health Study, and the Northern Sweden Health and Disease Study. These 3 publications reported data for total cancer mortality [48], colorectal cancer incidence [50], and esophageal cancer incidence [44]. Five publications were from the Danish Diet, Cancer and Health Study, also within HELGA, and examined colorectal [56], pancreatic [59], prostate [49], breast [55], and endometrial [54] cancer. One additional publication from within the HELGA cohort, the Norwegian Women and Cancer Study, published data on colorectal cancer in females [38]. Two separate publications within EPIC, but not part of HELGA, examined breast cancer [45] and total cancer [60] incidence. Despite the large percentage of studies within the EPIC cohort, possible overlap of study participants was only plausible for colorectal cancer [38,50,56,58]. Only 1 of these studies qualified for inclusion in the meta-analysis [38], and is discussed below in the section on meta-analysis results.

The type of bread consumed varied across studies. Bread was classified as nonwhite [48,58], dark [43,57], whole-grain [38,39,41,44,49,50,52,[54], [55], [56],^60^], whole wheat [40,46], wholemeal [42,47], whole-grain rye [49,51,56], rye [40,46,54,55,59], high-fiber or low-fiber [45], white [42,43,53,57,58], crisp [44,48,50], and “other” [60]. Only 2 studies reported data for total bread [37,58].

Study quality assessment is presented in Supplemental Table 2. Overall, 19 of the 24 studies received total scores between 7 and 9, and 5 received scores between 4 and 6.

### Systematic review

Data were most abundant for colorectal cancer (9 publications), breast cancer (6 publications), prostate cancer (4 publications), and total cancer mortality (4 publications). A total of 108 HRs, IRRs, MRRs, and RRs for cancer incidence or mortality were reported for bread consumption in highest compared with lowest (47 outcomes; Table 1) and dose-response analyses (61 outcomes; Table 2). Of these, 86 (79.6%) were not statistically significant (95%CI included 1.00), 11 (10.2%) indicated lower incidence or mortality associated with higher bread consumption, and 11 (10.2%) reported a greater incidence associated with higher bread consumption. Of the 11 instances showing increased cancer risk associated with higher bread intake, 9 were for white bread. Six of the 9 HRs for white bread came from 1 study that included both categorical and dose-response HRs for colorectal, colon, and rectal cancer [42].

### Total cancer

None of the studies reported a higher rate of total cancer mortality associated with bread intake in categorical or dose-response analyses [39,41,48,60], with 1 study indicating significantly lower risk. Among males in the HELGA cohort [48], the highest intake quartile for nonwhite bread consumption (median intake 194 g/d) was associated with a 21% lower rate of cancer mortality in males compared with males in the lowest quartile (13 g/d).

### Colorectal cancer

For colorectal cancer, a total of 48 outcomes were reported in categorical and dose-response analyses, including 8 that indicated lower incidence associated with higher bread consumption, and 8 that indicated greater incidence associated with higher bread intake (TABLE 1, TABLE 2). Among Norwegian females, consuming >180 g/d of whole-grain bread was associated with a 34% lower incidence of proximal colon cancer compared with not consuming whole-grain bread [38]. Among Swedish females, consuming >2 slices/d of whole-grain rye bread was associated with a 26% lower incidence of colorectal cancer compared with consuming <4 slices/wk [51].

Of the 32 outcomes reported in dose-response analyses, 6 indicated that increasing the intake of whole-grain [50,56], whole-grain rye [51], or nonwhite [58] bread by ∼0.5–1 slice/d was associated with a ∼4% to 12% lower incidence of colorectal cancer. Two of the studies reported HRs for total bread intake. Among Japanese males and females, the highest quartile of total bread intake (range = 45–720 g/d for males and 47–720 g/d for females) was not associated with colorectal cancer risk compared with the lowest quartile of total bread intake (0–4 g/d for both males and females) [37]. In the EPIC cohort, total bread consumption [per 1-SD/d increase] was not associated with colorectal cancer risk [58].

Three studies reported outcomes for white bread consumption. In the UK Biobank cohort [42], the highest tertile of white bread consumption was associated with a 22% higher incidence of colorectal and colon cancer and a 35% higher incidence of rectal cancer. In dose-response analyses, each 1-SD/d increase in white bread consumption was associated with a 10% higher incidence of colorectal cancer, a 9% higher incidence of colon cancer, and a 15% higher incidence of rectal cancer. In the EPIC cohort [58], each 1-SD/d increase in white bread consumption was associated with a 5% higher incidence of colorectal cancer (Table 2). In the Oxford Vegetarian Study [53], consuming 15+ slices/wk of white bread was associated with a 2.11-fold higher incidence of colorectal cancer compared with consuming <15 slices/wk.

### Breast cancer

For associations between breast cancer and bread intake, 5 studies (3 categorical and 2 dose-response) reported a total of 19 outcomes, 16 of which were not statistically significant (TABLE 1, TABLE 2). Among Swedish females in the Malmo Diet and Cancer cohort [45], the highest intake of high-fiber bread (median intake = 65 g/d) was associated with a 25% lower incidence of breast cancer compared with females who consumed no high-fiber bread, whereas the highest intake of low-fiber bread (median intake = 116 g/d) show no increased breast cancer incidence compared with the lowest intake of low-fiber bread (median intake = 18 g/d). By contrast, in the AGES-Reykjavik cohort, “daily or more” intake of rye bread was associated with an 80% higher incidence of breast cancer compared with “less than daily” intake [40]. Consumption of whole wheat bread in this cohort was not associated with breast cancer incidence (Table 1). In the Nurse’s Health Study II [57], premenopausal intake of white bread was associated with a 2% increased risk of breast cancer for each 2 servings/wk consumption, but this was only statistically significant for all cases combined and not for premenopausal and postmenopausal cases analyzed separately (Table 2). Adolescent intake of white or dark bread was not associated with breast cancer risk in this cohort.

### Prostate cancer

For prostate cancer, 14 of 15 outcomes indicated no significant associations between bread intake and prostate cancer incidence of mortality (TABLE 1, TABLE 2). Among middle-aged males in the NIH-AARP Diet and Health Study, self-reported recall of adolescent intake of dark bread ≥3 times/wk was associated with a 6% higher incidence of nonadvanced prostate cancer compared with adolescent intake <11 times/y [43]. In the only study that examined white bread, the consumption of white bread 2 times/d compared with ≤2 times/wk was not associated with prostate cancer incidence or mortality [43].

### Other cancers

Limited data were published for other site-specific cancers. In the HELGA cohort, esophageal cancer incidence was 12% lower per 25 g/d higher intake of whole-grain bread [44] (Table 2). Among Danish adults, a trend for lower pancreatic cancer incidence was observed for rye bread intake (HR: 0.89, 95% CI: 0.79, 1.00, per 50 g/d) [59] (Table 2). Bread consumption was not associated with ovarian [52], endometrial [54], stomach, or lung [47] cancers.

## Meta-Analyses

### Total cancer mortality

**FIGURE 2:**
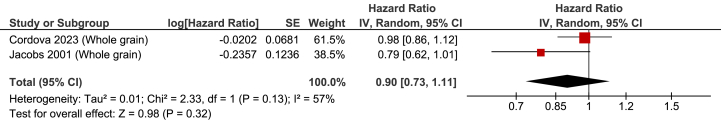
Forest plot of HRs for whole-grain bread consumption and cancer mortality, comparing highest vs. lowest intakes. CI, confidence interval; HR, hazard ratio.

Only 2 of the 4 publications that examined total cancer mortality reported HRs [39,41], and meta-analysis of these 2 studies revealed no association with whole-grain bread intake (HR: 0.90; 95% CI: 0.73, 1.11; *P* = 0.32; χ^2^ = 2.33, *P* = 0.13; *I*^2^ = 57%; Figure 2). Because of the small number of studies, no assessment of publication bias was performed.

### Supplemental analysis

Two additional publications included MRRs for wholemeal bread [47] and nonwhite and crisp bread [48]. The latter study included separate MRRs for males and females for both nonwhite and crisp bread. This supplemental meta-analysis with 7 studies indicated that the highest bread intake group was associated with a 10% lower total cancer mortality rate (μ = 0.90; 95% CI: 0.84, 0.96; *P* = 0.003; Supplemental Figure 1) with modest heterogeneity (χ^2^ = 6.68, *P* = 0.35; *I*^2^ = 10%). There was evidence of significant publication bias (Egger’s test *P* = 0.04).

### Site-specific cancer

**FIGURE 3:**
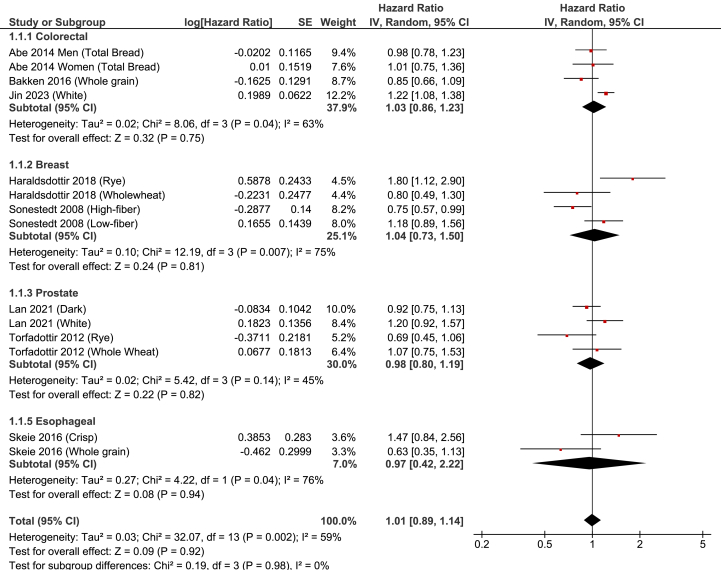
Forest plot of HRs for bread consumption site-specific cancer incidence, comparing highest vs. lowest intakes. CI, confidence interval; HR, hazard ratio.

Eight publications reported a total of 14 HRs for site-specific cancer, including 4 HRs each for colorectal cancer [37,38,42], breast cancer [40,45], and prostate cancer [43,46], and 2 for esophageal cancer [44]. Overall, the highest bread intake group was not associated with cancer incidence or mortality (HR: 1.01; 95% CI: 0.89, 1.14; *P* = 0.92; Figure 3). Heterogeneity was moderate-to-strong (χ^2^ = 32.07, *P* = 0.002; *I*^2^ = 59%). There was no evidence of publication bias (Egger’s test *P* = 0.79; symmetric funnel plot, Supplemental Figure 2).

### Supplemental analysis

Expanding the meta-analysis to also include 7 additional studies that reported MRRs, IRRs, and RRs did not change the result (μ = 1.00; 95% CI: 0.92, 1.09; *P* = 0.96), with moderate-to-strong heterogeneity (χ^2^ = 55.07, *P* = 0.0005; *I*^2^ = 51%) (Supplemental Figure 3). There was no evidence of publication bias (Egger’s test *P* = 0.78; symmetric funnel plot, Supplemental Figure 4).

### Colorectal cancer

Three publications reported 4 HRs for colorectal cancer incidence or mortality. Bread was reported as total bread [37], whole-grain [38], and white [42]. The highest bread intake group was not associated with colorectal cancer incidence (HR: 1.03; 95% CI: 0.86, 1.23; *P* = 0.75) (Figure 3). There was evidence of moderate-to-strong heterogeneity (χ^2^ = 8.06, *P* = 0.04; I^2^ = 63%). The significant heterogeneity was entirely because of the 1 study on white bread [42], as the removal of that study resulted in zero heterogeneity (χ^2^ = 0.96, *P* = 0.62; *I*^2^ = 0%).

### Supplemental analysis

The inclusion of MRRs, IRRs, and RRs from 4 additional studies [47,50,51,53] did not change the supplemental meta-analysis (μ = 0.97, 95% CI: 0.83, 1.13; *P* = 0.69; χ^2^ = 29.68, *P* = 0.002; *I*^2^ = 70%) (Supplemental Figure 3). There was no evidence of publication bias (Egger’s test *P* = 0.75). However, restricting the meta-analysis to only studies that examined bread other than white [37,38,47,50,53] resulted in a significantly lower incidence of colorectal cancer in the highest intake groups (μ = 0.87, 95% CI: 0.79, 0.96; *P* = 0.007; χ^2^ = 5.62, *P* = 0.59; *I*^2^ = 0%), with no evidence of publication bias (Egger’s test *P* = 0.41). And when restricting analysis to only the 4 studies that examined whole-grain [38,50,51] or wholemeal [47] bread, μ was lowered even further (μ = 0.83, 95% CI: 0.72; 0.97; *P* = 0.02; χ^2^ = 3.24, *P* = 0.36; *I*^2^ = 7%).

Two of the publications were from the HELGA cohort, including 1 with females in the Norwegian Women and Cancer Study [38] and 1 that included participants from all 3 Scandinavian cohorts within HELGA [50]. Thus, the possible effect of duplication of cancer cases existed. The removal of either or both of these studies did not change the result.

### Breast cancer

Four HRs from 2 publications were included in the meta-analysis for breast cancer incidence, with 1 reporting separate HRs for rye and wholewheat bread [40] and the other reporting separate HRs for high-fiber and low-fiber bread [45]. The highest bread consumption group was not associated with breast cancer incidence (HR: 1.04; 95% CI: 0.73, 1.50; *P* = 0.81), although there was strong heterogeneity (χ^2^ = 12.19, *P* = 0.007; *I*^2^ = 75%) (Figure 3).

### Supplemental analysis

The inclusion of 1 additional study that reported an MRR for wholemeal bread [47] did not change the result (μ = 1.07; 95% CI: 0.79, 1.45; *P* = 0.81; χ^2^ = 12.78, *P* = 0.01; *I*^2^ = 69%; Supplemental Figure 3).

### Prostate cancer

Four HRs from 2 publications were included in the meta-analysis for prostate cancer [43,46]. Bread types included whole wheat and rye [46] and dark and white [43]. The highest bread intake group was not associated with the risk of prostate cancer (HR: 0.98; 95% CI: 0.80, 1.19; *P* = 0.82) (Figure 3). There was modest-to-moderate heterogeneity (χ^2^ = 5.42, *P* = 0.14; *I*^2^ = 45%).

### Supplemental analysis

The inclusion of 2 additional publications that reported an MRR for wholemeal bread [47] or IRRs for whole-grain rye and whole-grain bread [49] did not change the result (μ = 0.99; 95% CI: 0.88, 1.10; *P* = 0.81; χ^2^ = 7.68, *P* = 0.26; *I*^2^ = 22%; Supplemental Figure 3), with no significant publication bias (Egger’s test *P* = 0.86).

### Other cancers

Only 3 publications provided data on other cancers. Whole-grain bread intake was not associated with ovarian cancer [52], whole-grain and crisp bread intake were not associated with esophageal cancer [44], and wholemeal bread intake was not associated with stomach cancer, pancreatic cancer, or lung cancer [47] (Table 1 and Supplemental Figure 3).

### Bread type

For overall site-specific cancers (Figure 3), to determine if bread type affected the results, we removed the 2 studies of white bread [42,43] and the 1 study of low-fiber bread [45]. The result of the meta-analysis was unchanged (HR: 0.93; 95% CI: 0.82, 1.07; *P* = 0.33), with modest-to-moderate heterogeneity (χ^2^ = 17.86; *P* = 0.06; *I*^2^ = 44%). After additional removal of the 2 HRs from 1 publication on total bread for males and females [37], the result also remained unchanged (HR: 0.92; 95% CI: 0.77, 1.10; *P* = 0.36), with moderate-to-strong heterogeneity (χ^2^ = 17.09, *P* = 0.03; I^2^ = 53%). Restricting the analysis to the 6 studies that examined whole-grain [38,44], wholewheat [40,46], high-fiber [45], or dark [43] bread resulted in a 14% lower cancer incidence (HR: 0.86; 95% CI: 0.76, 0.97; *P* = 0.02), with zero heterogeneity (χ^2^ = 4.00, *P* = 0.55; *I*^2^ = 0%) and no evidence of publication bias (Egger’s test *P* = 0.53). By contrast, restricting the analysis to the 3 studies that examined either white [42,43] or low-fiber [45] bread resulted in a 21% higher cancer incidence or mortality associated with the highest intakes (HR: 1.21; 95% CI: 1.09, 1.34; *P* = 0.0003), with zero heterogeneity (χ^2^ = 0.05, *P* = 0.97; *I*^2^ = 0%).

### Supplemental analyses

The inclusion of all 14 publications on site-specific cancer (Supplemental Figure 3) did not impact the results for bread. Restricting the meta-analysis to the 10 publications that examined whole-grain [38,44,[49], [50], [51], [52]], wholewheat [40,46], wholemeal [47], or high-fiber [45] bread also did not change the overall result (μ = 0.92; 95% CI: 0.84, 1.02; *P* = 0.10). Heterogeneity was not significant (χ^2^ = 19.64, *P* = 0.19; *I*^2^ = 24%), and there was no evidence of publication bias (Egger’s test *P* = 0.49). Meta-analysis of the 4 publications that examined rye bread [40,46,49,51] indicated no association with cancer incidence (μ = 0.92; 95% CI: 0.67, 1.26; *P* = 0.60; χ^2^ = 11.37, *P* = 0.01; *I*^2^ = 74%). The inclusion of 1 additional study that reported an RR for white bread [53] to the 3 that reported HRs for white [42,43] or low fiber [45] also did not change the result for white/low-fiber bread (μ = 1.24; 95% CI: 1.10, 1.39; *P* = 0.0004). Heterogeneity was modest (χ^2^ = 3.35, *P* = 0.34; *I*^2^ = 10%).

### Sensitivity analyses

We performed leave-one-out sensitivity analyses for all total and site-specific cancer outcomes, for both our primary analysis (studies reporting HRs) and supplemental analyses. In all instances, removal of any 1 study outcome did not change the results with regard to whether the highest bread consumption was associated with cancer risk (for HR or μ, all *P* > 0.05).

## Discussion

The results of this systematic review and meta-analysis show that bread consumption is not associated with increased cancer incidence or mortality. Nearly 90% (97 of 108) of the outcomes from the 24 cohort studies indicated either no association between bread consumption and cancer incidence or mortality, or a reduced incidence or mortality rate associated with bread higher intake. To our knowledge, our site-specific cancer meta-analysis is the first to show that bread consumption is not associated with risk for colorectal, breast, or prostate cancer. The 10% lower cancer mortality rate among those in the highest bread intake group for whole-grain or nonwhite bread (using all results from the supplemental analysis) is consistent with findings from previous meta-analyses that reported that whole-grain bread consumption was associated with an ∼10% to 15% lower risk of cancer mortality [[28], [29], [30]]. The observed inverse relationship may be attributable, in part, to the 16% lower colorectal cancer incidence we observed when restricting the meta-analysis for colorectal cancer to only those studies that assessed whole-grain or wholemeal bread.

The motivation for our review stemmed from the potential cancer risk associated with carcinogens in bread, especially acrylamide. Bread is a major source of dietary acrylamide because of its prevalent consumption worldwide [4]. This is especially relevant to the present review because bread is the greatest source of dietary acrylamide in the EPIC cohorts [61]. Of the 24 publications included in this review, 13 are from 1 or more of the EPIC cohorts [38,39,44,45,[48], [49], [50],[54], [55], [56],[58], [59], [60]]. Regarding dietary acrylamide exposure and cancer risk, results from cohort studies are inconclusive. Some meta-analyses have reported higher cancer risk [11,14,15] whereas several other systematic reviews and meta-analyses demonstrated that most cohort studies show no increased cancer risk associated with dietary acrylamide exposure [12,13,16,17]. Thus, despite having been determined to be “probably carcinogenic to humans” by the IARC in 1994 [7] and “reasonably anticipated to be a human carcinogen” according to the NTP’s report on carcinogens [8], published data on dietary acrylamide intake and cancer risk in humans suggest that risks are low. Our results strongly suggest that this is true specifically with regard to bread consumption.

Observations in multiple cohorts consistently show whole grains are associated with a lower risk of cancer [[62], [63], [64]], especially colorectal cancer [62]. Whole grains are an important source of cereal fiber, which is also associated with the reduced risk of cancer, and this may account for the lower cancer risk associated with whole and total grain intake [65]. Interestingly, acrylamide concentrations in whole-grain bread and related foods are generally higher than white bread [66], most likely because whole-grain bread has higher concentrations of acrylamide-precursor asparagine [67,68]. Despite higher acrylamide concentrations, whole-grain foods are major sources of antioxidants, phenolics, and other bioactive compounds, which reduce oxidative damage may provide anticarcinogenic properties [[69], [70], [71]]. Fiber from bread can increase production of short-chain fatty acids, such as butyrate. Butyrate is a major energy source for human colon cells [72], and has been reported to inhibit growth of cancerous cells, mainly by inducing apoptosis [73], and is protective against colorectal cancer [74,75].

Whether results would have differed if non-whole-grain bread had been the primary bread that was examined is unclear, because only a few studies included in our review examined bread other than whole grain. Total bread intake was not associated with colorectal cancer risk in Japanese [37] or EPIC [58] cohorts. In the EPIC cohort, the consumption of carbohydrate foods was associated with reduced risk of breast cancer, and bread contributes the highest proportion of carbohydrates in that cohort [76]. Low-fiber bread was not associated with breast cancer risk in the Malmo Diet and Cancer cohort [45]. White bread intake was associated with higher incidence of colorectal cancer in the UK Biobank [42] and EPIC [58] cohorts, and in the Oxford Vegetarian Study [53], and higher incidence of breast cancer in the Nurses’ Health Study II cohort [57]. Within the latter cohort, the higher risk of breast cancer was modest (2% increase per 2 servings/wk of white bread) and was statistically significant for premenopausal white bread intake only when both premenopausal and postmenopausal breast cancer cases were combined. Neither adolescent nor premenopausal white bread intake was associated with premenopausal or postmenopausal breast cancer incidence. White bread was not associated with prostate cancer incidence or mortality in the NIH-AARP Diet and Health Study [43]. Although our meta-analysis of the 4 studies that examined either white [42,43,53] or low-fiber [45] bread indicated a 24% higher cancer risk in the highest intake groups, this result should be viewed with caution. One of the 3 studies relied on recall of adolescent food intake among adults aged 50–70 y [43]. Although adolescent diet is captured reasonably well with FFQs in young adults [77], this is not the case for older adults [78]. Another study was judged to be of relatively low quality due mainly to limited adjustments in the statistical model, raising the possibility of significant residual confounding [53]. More research on the association between white bread consumption and cancer risk is warranted.

As mentioned above, acrylamide concentrations in white bread are generally lower than those in whole-grain bread. Thus, if the consumption of white bread increases cancer risk, it is not likely attributable to acrylamide. The generally higher GI of white bread may be a contributing factor, as several meta-analyses have shown that high-GI diets are associated with increased cancer risk [[20], [21], [22], [23], [24]]. However, white bread consumption is frequently associated with overall low diet quality [79,80], including intake of foods that have been shown to be associated with increased cancer risk, such as red and processed meat [[81], [82], [83], [84]]. This latter point is especially relevant to United States Dietary Guidelines regarding dietary patterns. The 2020–2025 United States Dietary Guidelines focuses on the importance of encouraging healthy dietary patterns at every stage of life [85], and the Dietary Guidelines Advisory Committees rely heavily on studies that examined dietary patterns, and not separate foods [86,87]. The most frequent foods contributing to healthy dietary patterns are vegetables, fruits, whole grains, low-fat or nonfat dairy, seafood, legumes, nuts, and beans [[88], [89], [90]]. By contrast, unhealthy dietary patterns include red and processed meat, sugar-sweetened foods and beverages, French fries, high-fat dairy products, and refined grains [[88], [89], [90]]. Epidemiologic evidence indicates that healthy dietary patterns are associated with a lower risk of cancer whereas unhealthy dietary patterns are associated with increased risk of cancer [[88], [89], [90], [91]].

Our findings on whole-grain bread support the inclusion of whole-grain foods within a healthy dietary pattern. Within the unhealthy dietary pattern, however, it is primarily red and processed meat [[81], [82], [83], [84]], and sugar-sweetened beverages [[92], [93], [94]] that are most consistently shown to be associated with increased cancer risk. The evidence for an association between cancer risk and refined grain intake (which includes white and low-fiber bread) is limited and inconsistent [28,62,83,95]. Moreover, research on refined grain intake and health outcomes is confounded by the fact that most studies that examined refined grain intake separately from an unhealthy dietary pattern have defined refined grains to include both staple refined grain foods such as breads, cereals, and pasta, and indulgent refined grain foods such as cookies, cakes, donuts, muffins, sweet rolls, and pizza [95]. This greatly reduces the ability to quantify cancer risk of a specific food (e.g., white bread) within such a heterogeneous category like refined grains. Nonetheless, it is worth noting that current 2020–2025 United States Dietary Guidelines include the recommendation to “make half your grains whole grains,” and acknowledges that refined grains (<3 servings/d) can be a part of a healthy United states-style dietary pattern [85]. Scientific support for the “make half your grains whole grains” recommendation is well documented [96].

Our categorical meta-analysis was intended to examine whether high intakes of bread were associated with increased risk of cancer. Not all studies reported the quantity of bread consumed in the highest and lowest intake groups. But among those that did, the amount of bread in the highest intake group varied considerably across studies (Table 1). The highest intake groups in several studies exceeded 100 g/d, with some ∼150 to 200 g/d [38,44,45,48,50]. Although there is no standard value for the weight of a slice of bread, it is generally in the range of 30–50 g. Thus, the highest intake groups in these studies could have consumed ∼2 to 7 slices of bread per day. In the National Health Screening Study of Norway, bread consumption in the highest intake groups for males and females was >7 slices/d [41]. Yet, in each of these studies, not a single IRR or MRR indicated a significantly higher cancer risk in the highest intake group.

### Strengths and Limitations

With few exceptions, the HRs reported for categorical and dose-response analyses indicated no increased cancer risk associated with bread consumption, and the highest categories of bread consumption were actually associated with a lower risk of cancer mortality. There was no evidence of publication bias in any of the meta-analyses, and study quality was rated as high in 19 of the 24 publications included in our review, and moderate in the other 5. Bread consumption in the highest intake groups was ∼2 to 7 slices/d, which suggests that even at these relatively high consumption levels cancer risk was not evident.

Nevertheless, limitations of nutritional epidemiology must be acknowledged [97,98], especially with regard to cancer [99]. In most studies, dietary intake was assessed with the FFQ at one point in time. Although FFQs have been validated, their use in epidemiologic studies has been questioned [100,101]. Thus, there is potential for residual confounding because of measurement error. Although the HRs from the cohort studies included in this review are from fully adjusted statistical models, confounding from unmeasured variables is likely [102]. Several examples are worth noting. Only 2 studies included adjustments for dietary fiber [37,38]. Dietary fiber, especially cereal fiber, is associated with reduced cancer risk [63,65]. Eleven of the studies did not include adjustment for physical activity [38,40,44,[46], [47], [48],[50], [51], [52], [53], [54]], which is associated with a lower risk of cancer [103]. None of the studies included adjustment for GI, which been reported in several meta-analyses to be associated with increased cancer risk [[20], [21], [22], [23], [24]]. Other dietary variables such as low diet quality for diets high in white bread may also be operative. Overall, probable confounding from unmeasured variables likely diminishes the quality of evidence.

Most of the studies defined bread as whole grain, wholemeal, or nonwhite. Some studies included rye bread, although it was not always clear whether the rye bread was whole grain. Only 4 studies included white bread, which represents the major source of bread consumption in most Western countries. Thus, the current findings cannot easily be extended to white bread consumption.

The majority of studies were from European countries, which may not be representative of bread consumption (quantity and type) in other countries. Even the EPIC cohort may not be nationally representative samples of the European general population [61]. Only 2 United States cohorts and 1 Asian cohort were included in the systematic review, and the meta-analysis included only 1 non-European cohort.

### Conclusions

The results of this systematic review and meta-analysis indicate that bread consumption is not associated with increased site-specific cancer risk. Thus, it is likely that heat toxins produced in bread in the amounts customarily eaten have little or no impact on cancer incidence or mortality. High whole-grain bread consumption is associated with a lower risk of total cancer mortality and colorectal cancer incidence.

## Author contributions

The authors’ contributions were as follows – GAG: design and conception of the study, development of overall research plan, and study oversight; GAG, SSA: performed literature search; GAG, SSA, CP: selected eligible studies; GAG: performed meta-analyses; GAG, SSA, JMJ: wrote paper; GAG, SSA, JMJ, CP: had primary responsibility for final content; and GAG, SSA, JMJ, CP: read and approved the final manuscript.

## Conflicts of interest

GAG, SSA, and JMJ are members of the Scientific Advisory Board of the Grain Foods Foundation. SSA is a member of the Scientific Advisory Board of the Alliance for Potato Research and Education. The other author reports no conflicts of interest.

## Data availability

The authors confirm that the data supporting the findings of this study are available with the article and its supplemental materials.

## Funding

This work was sponsored by the Grains Food Foundation. The sponsor had no involvement in study design; collection, analysis, and interpretation of data; writing of the manuscript; or restrictions regarding publication.
