# Supplementary material for: Bread Consumption and Cancer Risk: Systematic Review and Meta-Analysis of Prospective Cohort Studies
Source: Curr Dev Nutr. 2024 Nov 2;8(12):104501. doi: 10.1016/j.cdnut.2024.104501 (PMC11634998; doi:10.1016/j.cdnut.2024.104501)
Supplement: multimedia component 1 [file mmc1.pdf]

Bread consumption and cancer risk: Systematic review and meta-analysis of prospective cohort studies
GA Gaesser

Supplemental Figure 1

Forest plot of risk ratios for bread consumption and cancer mortality, comparing highest vs. lowest intakes. HR, Hazard Ratio; HHR, Hazard Rate Ratio; MMR, Mortality Rate Ratio; μ, pooled risk ratio; CI, confidence interval

Study or Subgroup	μ	SE	Weight	μ IV, Random, 95% CI
Appleby 2002 (Wholemeal) - MRR	0.01	0.0879	15.1%	0.01 [-0.16, 0.18]
Cordova 2023 (Whole grain) - HR	-0.0202	0.0681	23.4%	-0.02 [-0.15, 0.11]
Jacobs 2001 (Whole grain) - HRR	-0.2357	0.1236	8.0%	-0.24 [-0.48, 0.01]
Johnsen 2015 Men (Crisp) - MRR	-0.1863	0.1101	10.0%	-0.19 [-0.40, 0.03]
Johnsen 2015 Men (Non-white) - MRR	-0.2357	0.1047	11.0%	-0.24 [-0.44, -0.03]
Johnsen 2015 Women (Crisp) - MRR	-0.1393	0.0812	17.3%	-0.14 [-0.30, 0.02]
Johnsen 2015 Women (Non-white) - MRR	-0.1165	0.0873	15.2%	-0.12 [-0.29, 0.05]
**Total (95% CI)**			**100.0%**	**-0.11 [-0.18, -0.04]**

Heterogeneity: Tau² = 0.00; Chi² = 6.68, df = 6 (P = 0.35); I² = 10%
Test for overall effect: Z = 3.00 (P = 0.003)

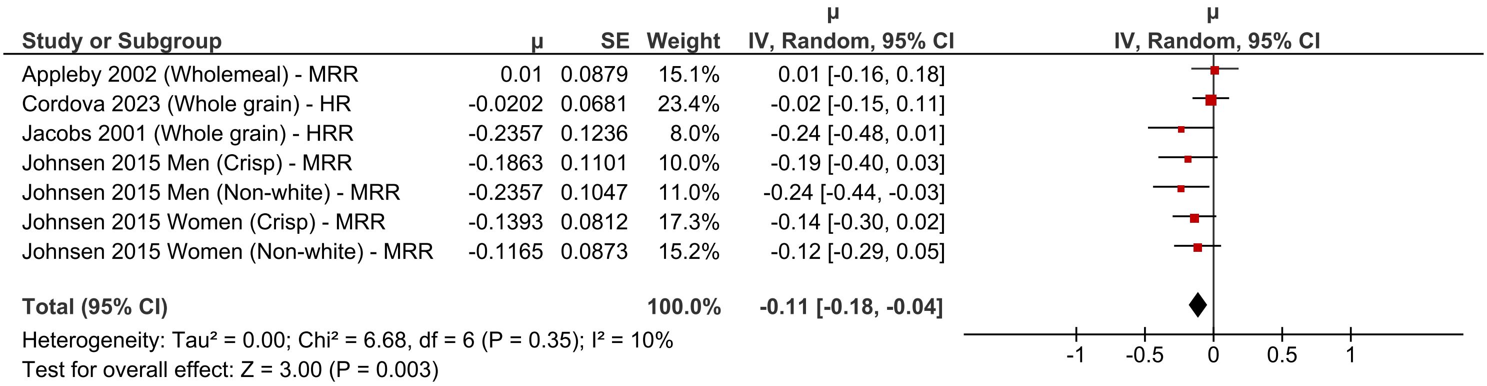

Supplemental Figure 2

Funnel plot for studies included in the meta-analysis of bread intake and site-specific cancer risk (Figure 3 in main manuscript). SE, Standard Error. Egger's test P = 0.79.

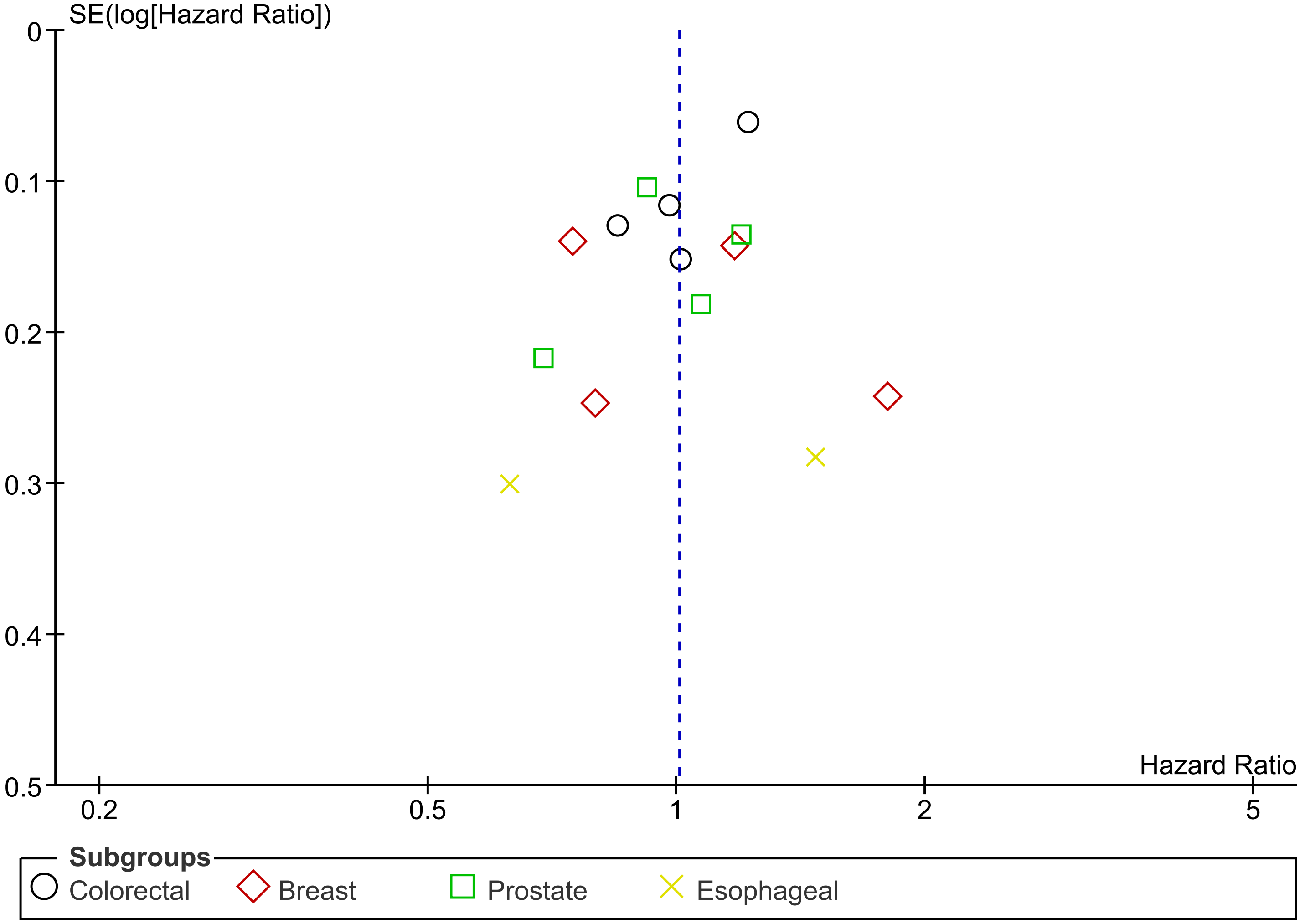

Supplemental Figure 3

Forest plot of risk ratios for bread consumption and site-specific cancer incidence or mortality, comparing highest vs. lowest intakes. HR, Hazard Ratio; HHR, Hazard Rate Ratio; IRR, Incident Rate Ratio, MMR, Mortality Rate Ratio; RR, Relative Risk; μ, pooled risk ratio; CI, confidence interval

Study or Subgroup	μ	SE	Weight	μ IV, Random, 95% CI
**1.1.1 Colorectal**				
Abe 2014 Men (Total Bread) - HR	-0.0202	0.1165	5.2%	-0.02 [-0.25, 0.21]
Abe 2014 Women (Total Bread) - HR	0.01	0.1519	4.2%	0.01 [-0.29, 0.31]
Appleby 2002 (Wholemeal) - MRR	0.1906	0.2373	2.5%	0.19 [-0.27, 0.66]
Bakken 2016 (Whole grain) - HR	-0.1625	0.1291	4.8%	-0.16 [-0.42, 0.09]
Jin 2023 (White) - HR	0.1989	0.0622	7.0%	0.20 [0.08, 0.32]
Kyro 2013 (Crisp) - IRR	-0.2107	0.1176	5.2%	-0.21 [-0.44, 0.02]
Kyro 2013 (Whole grain) - IRR	-0.2107	0.1176	5.2%	-0.21 [-0.44, 0.02]
Larsson 2005 (Whole grain Rye) - IRR	-0.3011	0.1433	4.4%	-0.30 [-0.58, -0.02]
Sanjoaquin 2004 (Brown bread) - RR	-0.1054	0.2181	2.8%	-0.11 [-0.53, 0.32]
Sanjoaquin 2004 (White bread) - RR	0.7467	0.3009	1.8%	0.75 [0.16, 1.34]
**Subtotal (95% CI)**			**43.0%**	**-0.03 [-0.18, 0.12]**
Heterogeneity: Tau² = 0.04; Chi² = 29.68, df = 9 (P = 0.0005); I² = 70%				
Test for overall effect: Z = 0.40 (P = 0.69)				
**1.1.2 Breast**				
Appleby 2002 (Wholemeal) - MRR	0.1989	0.2482	2.3%	0.20 [-0.29, 0.69]
Haraldsdottir 2018 (Rye) - HR	0.5878	0.2433	2.4%	0.59 [0.11, 1.06]
Haraldsdottir 2018 (Wholewheat) - HR	-0.2231	0.2477	2.3%	-0.22 [-0.71, 0.26]
Sonestedt 2008 (High-fiber) - HR	-0.2877	0.14	4.5%	-0.29 [-0.56, -0.01]
Sonestedt 2008 (Low-fiber) - HR	0.1655	0.1439	4.4%	0.17 [-0.12, 0.45]
**Subtotal (95% CI)**			**16.0%**	**0.07 [-0.24, 0.37]**
Heterogeneity: Tau² = 0.08; Chi² = 12.78, df = 4 (P = 0.01); I² = 69%				
Test for overall effect: Z = 0.44 (P = 0.66)				
**1.1.3 Prostate**				
Appleby 2002 (Wholemeal) - MRR	0.2151	0.3718	1.2%	0.22 [-0.51, 0.94]
Egeberg 2011 (Whle grain rye) - IRR	-0.1165	0.0987	5.8%	-0.12 [-0.31, 0.08]
Egeberg 2011 (Whole grain) - IRR	0.0677	0.0914	6.0%	0.07 [-0.11, 0.25]
Lan 2021 (Dark) - HR	-0.0834	0.1042	5.6%	-0.08 [-0.29, 0.12]
Lan 2021 (White) - HR	0.1823	0.1356	4.6%	0.18 [-0.08, 0.45]
Torfadottir 2012 (Rye) - HR	-0.3711	0.2181	2.8%	-0.37 [-0.80, 0.06]
Torfadottir 2012 (Whole Wheat) - HR	0.0677	0.1813	3.5%	0.07 [-0.29, 0.42]
**Subtotal (95% CI)**			**29.6%**	**-0.01 [-0.13, 0.10]**
Heterogeneity: Tau² = 0.00; Chi² = 7.68, df = 6 (P = 0.26); I² = 22%				
Test for overall effect: Z = 0.23 (P = 0.81)				
**1.1.4 Ovarian**				
Hedelin 2011 (Whole grain) - RR	0.392	0.2262	2.7%	0.39 [-0.05, 0.84]
**Subtotal (95% CI)**			**2.7%**	**0.39 [-0.05, 0.84]**
Heterogeneity: Not applicable				
Test for overall effect: Z = 1.73 (P = 0.08)				
**1.1.5 Esophageal**				
Skeie 2016 (Crisp) - HR	0.3853	0.283	1.9%	0.39 [-0.17, 0.94]
Skeie 2016 (Whole grain) - HR	-0.462	0.2999	1.8%	-0.46 [-1.05, 0.13]
**Subtotal (95% CI)**			**3.7%**	**-0.03 [-0.86, 0.80]**
Heterogeneity: Tau² = 0.27; Chi² = 4.22, df = 1 (P = 0.04); I² = 76%				
Test for overall effect: Z = 0.08 (P = 0.94)				
**1.1.6 Pancreatic**				
Appleby 2002 (Wholemeal) - MRR	-0.1508	0.3566	1.3%	-0.15 [-0.85, 0.55]
**Subtotal (95% CI)**			**1.3%**	**-0.15 [-0.85, 0.55]**
Heterogeneity: Not applicable				
Test for overall effect: Z = 0.42 (P = 0.67)				
**1.1.7 Stomach**				
Appleby 2002 (Wholemeal) - MRR	-0.1744	0.3537	1.3%	-0.17 [-0.87, 0.52]
**Subtotal (95% CI)**			**1.3%**	**-0.17 [-0.87, 0.52]**
Heterogeneity: Not applicable				
Test for overall effect: Z = 0.49 (P = 0.62)				
**1.1.8 Lung**				
Appleby 2002 (Wholemeal) - MRR	0.077	0.2492	2.3%	0.08 [-0.41, 0.57]
**Subtotal (95% CI)**			**2.3%**	**0.08 [-0.41, 0.57]**
Heterogeneity: Not applicable				
Test for overall effect: Z = 0.31 (P = 0.76)				
**Total (95% CI)**			**100.0%**	**-0.00 [-0.09, 0.08]**
Heterogeneity: Tau² = 0.03; Chi² = 58.01, df = 27 (P = 0.0005); I² = 53%				
Test for overall effect: Z = 0.09 (P = 0.93)				
Test for subgroup differences: Chi² = 3.93, df = 7 (P = 0.79), I² = 0%				

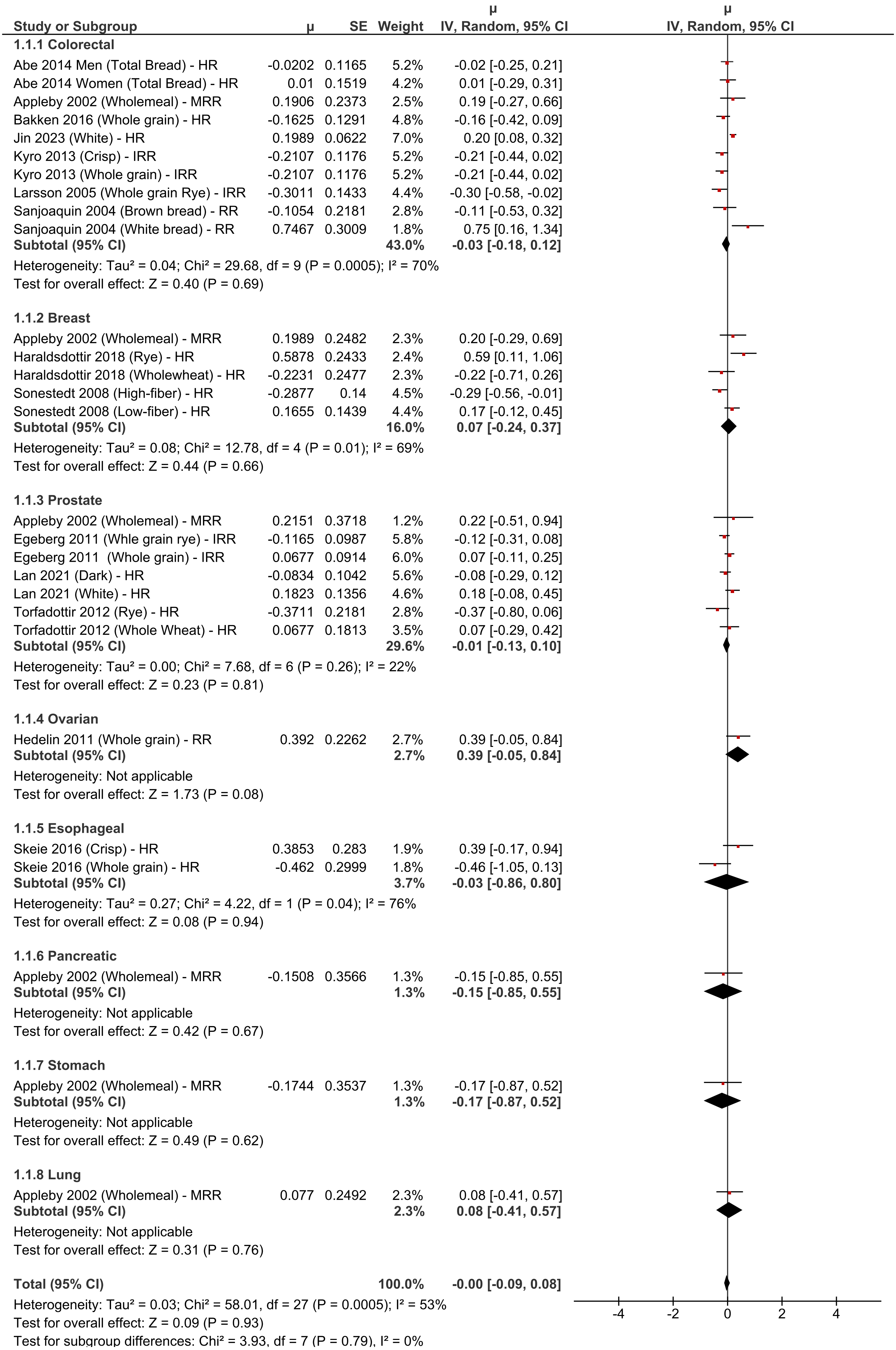

Supplemental Figure 4

Funnel plot for studies included in the meta-analysis of bread intake and site-specific cancer risk (Supplemental Figure 3). SE, Standard Error. Egger's test P = 0.78.

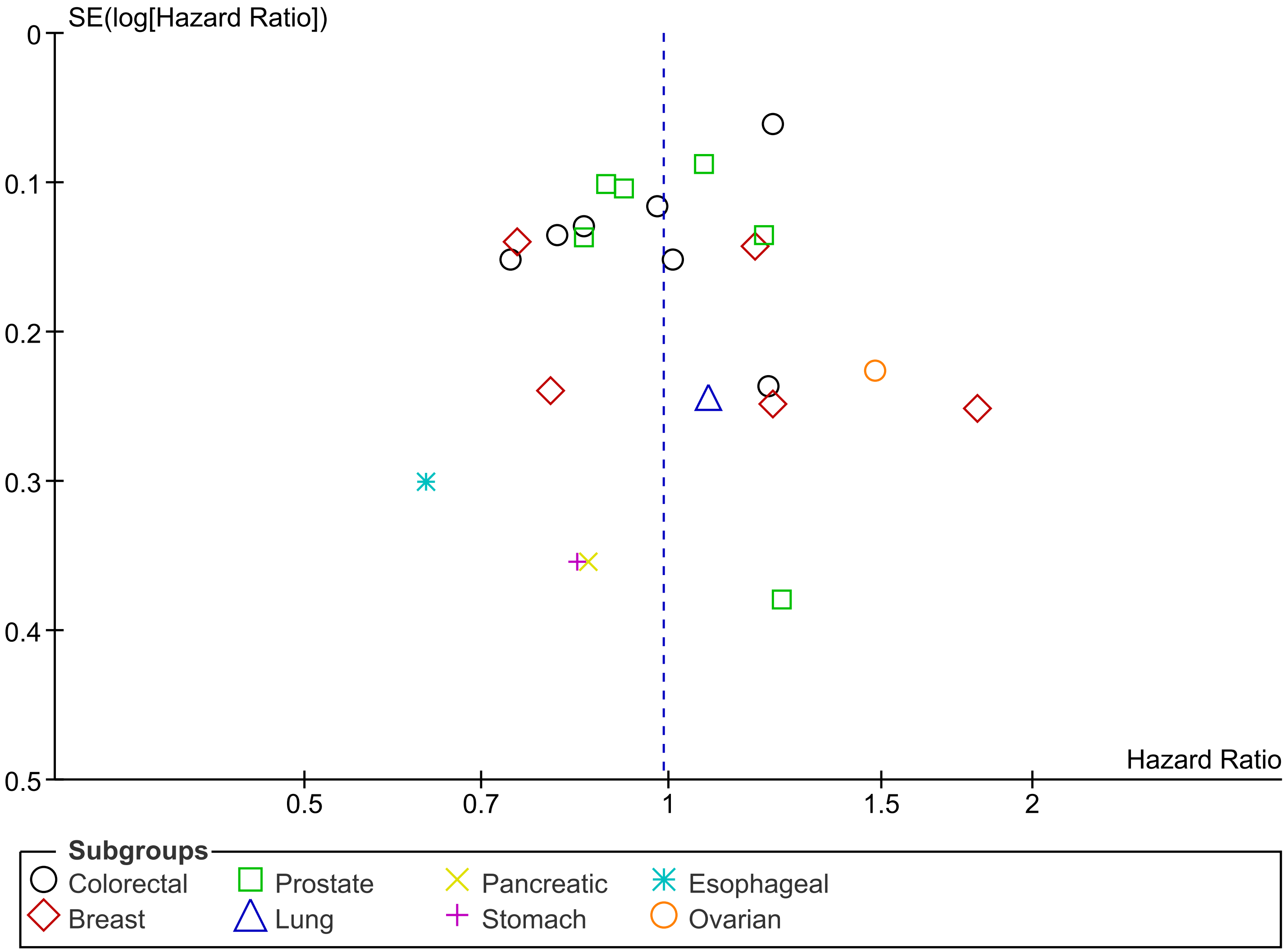

Supplemental Table 1. Description of cohort studies

Author	Cohort	Cohort Size	Age (yr)	Follow-up (years)	Dietary Assessment	Bread type	Adjustments in Statistical Model	Outcomes
Aarestrup 2012	Diet, Cancer and Health [Denmark]	24,418 women	50-64	13.5 (median)	192-item FFQ	Whole-grain bread<br>Rye bread	Menopausal status; use of hormone replacement therapy; type of HRT; parity; body mass index; smoking; energy intake; mutual adjustment for other whole-grain products (rye bread, whole-grain bread, oatmeal or muesli, crispbread)	Endometrial cancer incidence [217 cases]<br><br>Incident Rate Ratio: Dose-response: Per 50g/day increase
Abe 2014	Japan Public Health Center-based prospective Study [Japan]	34,559 men<br>38,942 women	45-74	Follow-up from 1995-1998 through 2008	FFQ	Total bread	Age; geographical area; smoking; alcohol; physical activity; history of diabetes; menopausal status; exogenous female hormones; total energy intake; intakes of red meat, calcium, magnesium, vitamin B6, vitamin B12, folate, vitamin D, polyunsaturated fatty acids, fiber, rice, noodles	Colorectal cancer incidence [777 cases for men; 499 cases for women]<br><br>Hazard Ratio: Quartile 4 (median intake = 60 g/d for both men and women) vs. Quartile 1 (median intake = 4 g/d for both men and women)
Anderson 2020	Diet, Cancer and Health [Denmark]	1,965 women	54-77	Follow-up from 1993-1997 through 2013	FFQ	Whole-grain bread<br>Rye bread	Age at diagnosis; year at diagnosis and time of follow-up since diagnosis; alcohol; smoking; physical activity; body mass index; education; adjustment for clinical variables as	Breast cancer mortality [301 cases]<br><br>Hazard Ratio (for pre-diagnosis intake): Dose-response: For whole-grain bread: “per 40g/day” increase

							tumor size, nodal status, and ER status	For rye bread: “per 50g/day increase”
Appleby 2002	Health Food Shoppers study [United Kingdom]	4,325 men 6416 women	16-89	Follow-up from 1973-1979 through 1997	Simple diet and lifestyle questionnaire	Wholemeal	Age; sex; smoking; fresh fruit; nuts/dried fruit; bran cereals	Mortality from all malignant neoplasms (680 cases); stomach cancer (40 cases); colorectal cancer (100 cases), pancreatic cancer (39 cases); lung cancer (81 cases); breast cancer (90 cases); prostate cancer (41 cases)<br><br>Death Rate Ratio: “Daily” vs. “less than daily”
Bakken 2016	Norwegian Women and Cancer (NOWAC Study) [Norway]	78,254 women	46-76	9	FFQ	Whole grain	Age; smoking; body mass index; hormone replacement therapy; intake of alcohol, red meat, processed meat, fiber from foods other than whole-grain bread, calcium from food; total energy intake	Colorectal, colon (proximal and distal), and rectal cancer incidence<br>Cases:<br>Colorectal: 795<br>Proximal: 316<br>Distal: 193<br>Rectal: 218<br><br>Hazard Ratio: Quartile 4 vs. Quartile 1
Cordova 2023	European Prospective Investigation into Cancer and Nutrition [Italy, Spain, United Kingdom, Netherlands, Germany, Sweden, Denmark]	106,116 men 160,550 women	35-74	11.1	Country-specific FFQ	Whole grain	Age; sex; educational level; marital status; smoking; leisure-time physical activity; diabetes; cardiovascular disease history; musculoskeletal disease; chronic lung disease; cancer history; depression; fresh fish; Cod; red meat	Cancer mortality (1,814 cases)<br><br>Hazard Ratio: “Daily” vs. “Less than Weekly”

							and pork products; dairy; legumes and vegetables; vegetable soup; potatoes; wine	
Egeberg 2010	Diet, Cancer and Health [Denmark]	26,630 men 29,189 women	50-64	10.6	192-item FFQ	Whole-grain bread Whole-grain rye bread	Body mass index; education; leisure-time physical activity; hormone replacement therapy (women); intakes of alcohol, red and processed meat; mutual adjustment for whole-grain bread, whole-grain rye bread, and oatmeal	Colon cancer and rectal cancer incidence<br>Cases (Colon):<br>Men: 244<br>Women: 217<br>Cases (Rectal):<br>Men: 169<br>Women: 114<br><br>Incident Rate Ratio separately for men and women:<br>Dose-response: Per 25g/day increment
Egeberg 2011	Diet, Cancer and Health [Denmark]	26,630 men	50-64	12.4	192-item FFQ	Whole-grain bread Whole-grain rye bread	Age; height; weight; smoking; red and processed meat; dairy products; sports activities; total energy intake; education	Prostate cancer incidence [1,081 cases]<br><br>Incident Rate Ratio:<br>Tertile 3 vs.Tertile 1<br>Dose-response: "per 25-g/day"
Farvid 2016	Nurses' Health Study II [United States]	90,516 women	27-44	22	~130-item FFQ for adult intake<br><br>124-item for recall of adolescent intake (n = 44,263)	Dark bread<br>White bread	Age; smoking; race (white, nonwhite); parity and age at first birth; height; body mass index at 18 years; weight change since age 18; age at menarche; family history of breast cancer; history of benign breast disease; oral contraceptive use; physical activity;	Breast cancer incidence [3,235 cases]<br><br>Relative Risk:<br>Dose-response:<br>"per 2 servings/week"

							adult alcohol intake; energy intake; hormone use in postmenopausal women; age at menopause	
Haraldsdottir 2018	Age Gene Environment Susceptibility (AGES)-Reykjavik [Iceland]	3,326 women	77 ± 6 (mean age and SD at entry)	8.8	FFQ Assessment of diet in adolescence (14-19 years), midlife (40-50 years), and late life (at time of entry)	Whole wheat<br>Rye	Age; education; body mass index; alcohol consumption in midlife; age at first child and age at menarche; intakes of meat, milk, oatmeal, fish, (whole wheat or rye bread)	Breast cancer incidence [97 cases]<br><br>Hazard Ratio: “Daily or more” vs. “less than daily” for adolescent and midlife intake
Hedelin 2011	Scandinavian Women’s Lifestyle and Health Cohort (WLH Study) [Sweden]	47,100 women	3-49	16	~80-item FFQ	Whole grain	Age; energy intake; BMI; education; country of birth; smoking; use of oral contraceptives; age at menarche; parity; use of postmenopausal hormone replacement therapy; dietary intake of alcohol, saturated fat, meat, and fish; nutrient densities	Ovarian cancer incidence [163 cases]<br><br>Hazard Ratio: Quartile 4 vs Quartile 1
Jacobs 2001	National Health Screening Service of Norway	16,933 men<br>16,915 women	35-56	Follow-up from 1977-1983 through 1994	66-item FFQ	Whole grain	Age; sex; energy intake; smoking; physical activity during leisure; physical activity during work; customary use of cod liver oil; customary use of multivitamins; saturated fat intake; systolic blood pressure; serum	Cancer mortality [843 cases]<br><br>Hazard Rate Ratio for Whole-grain bread score:<br>2.25 – 5.4 (~9 slices/d) vs. ≤0.60 (~1 slice/d)

							total cholesterol;<br>body mass index.	
Jin 2023	UK Biobank	118,200 men (44.6%) and women (55.4%)	55.9 ± 7.8 (mean ± SD)	12.8	UK Biobank 24h online touchscreen diet assessment questionnaire	White<br>Wholemeal	Age; sex; Townsend deprivation index; education; ethnicity; family history of colorectal cancer; regular aspirin use; bowel screening; diabetes; BMI; physical activity; smoking; total energy intake	Colorectal, Colon, and rectal cancer incidence<br>Cases:<br>Colorectal: 1,466<br>Colon: 842<br>Rectal: 359<br><br>Hazard Ratio:<br>Tertile 3 vs. Tertile 1<br><br>Dose-response: per SD
Johnsen 2015	Scandinavian HELGA cohort [Norway, Sweden, Denmark]	Norway: 37,111 women<br>Sweden: 12,348 men<br>13,203 women<br>Denmark: 27,069 men<br>29,787 women	30-64	Norway: 11.1<br>Sweden: 14.2<br>Denmark: 11.9	Norway: 88-item FFQ<br>Sweden: 98-item FFQ<br>Denmark: 173-item FFQ	Non-white	Age; smoking; follow-up time; education; alcohol; body mass index; total energy intake; whole-grain products and whole-grain types	Cancer mortality [1,375 cases for men; 1,775 cases for women]<br><br>Mortality Rate Ratio: Quartile 4 (median intake = 201 g/d for men, 180 g/d for women) vs. Quartile 1 (median intake = 64 g/d for men; 56 g/d for women)<br><br>Dose-response "per doubling of intake"
Kyro 2013	Scandinavian HELGA cohort [Norway, Sweden, Denmark]	38,841 men<br>69,159 women	30-64	11	FFQ	Whole grain	Age; smoking; education; hormone replacement therapy; body mass index; total energy intake; intakes of alcohol, red and processed meat	Colorectal cancer incidence [1,123 cases]<br><br>Incident Rate Ratio: Quartile 4 vs. Quartile 1<br>Dose-response "per 25 g/day increase"
Lan 2021	NIH-AARP Diet and Health Study [United States]	150,671 men	50-70	1,729,896 person-years of follow-up	37-item FFQ that assessed participant's diet at ages 12-13	Adolescent intake:<br>Dark bread<br>White bread	Age; race/ethnicity; education; marital status; smoking; waist circumference;	Prostate cancer incidence and mortality

							height; father's occupation; family history of prostate cancer; screening prostate-specific antigen and digital rectal examination; diabetes history; adolescent energy intake; adult alcohol intake; adolescent and adults body mass index; adolescent and adult physical activity; adult dietary intake of exposures of interest; adolescent intake of red meat and diary	Cases:<br>14,238 nonadvanced<br>2,170 advanced<br>760 deaths<br><br>Hazard Ratio:<br>Dark bread:<br>≥3 times/wk vs. <11 times/year<br><br>White bread:<br>2/day vs. ≤2 times/wk
Larsson 2005	Swedish Mammography Cohort [Sweden]	61,433 women	40-76	14.8	67-item FFQ	Hard whole grain rye bread	Age; body mass index; education; total energy intake; intakes of saturated fat, calcium, red meat, fruits, vegetables.	Colorectal cancer incidence [805 cases]<br><br>Incident Rate Ratio:<br>≥2 slices/day vs. <4 slices per week;<br><br>Dose-response: "per 1 slice/day increase"
Papadimitriou 2022	European Prospective Investigation into Cancer and Nutrition (EPIC) [Denmark, France, Germany, Greece, Italy, Netherlands, Norway, Spain, Sweden, United Kingdom]	EPIC: 112,788 men 274,004 women<br><br>NLCS: 4,023 men 3473 women	35-70	14.1	FFQ (country specific)	Non-white bread<br>White bread<br>Total bread	Age; sex; smoking; body mass index; physical activity; diabetes history; total energy intake; education; family history of colorectal cancer;	Colorectal cancer, colon cancer and rectal cancer incidence<br><br>Cases:<br>EPIC: 5,069 (56.8% women)<br>Colon: 3,143 (1,495 proximal, 1,435 distal, 213 unspecified)<br>Rectal: 1,715<br><br>NLCS: 3,765 (42.8% women)

	Netherlands Cohort Study (NLCS) [Netherlands]							Colon: 2,612 (1,348 proximal, 1,187 distal)<br>Rectal: 801<br><br>Hazard Ratio: Dose-response: per 1 SD/day increase consumption
Sanjoaquin 2004	Oxford Vegetarian Study [United Kingdom]	4,162 men<br>6,836 women	16-89	17	FFQ	Brown bread<br>White bread	Age; sex; smoking; alcohol	Colorectal cancer incidence [95 cases]<br><br>Relative risk for "≥15 slices per week" vs. "<15 slices per week"
Schacht 2021	Diet, Cancer and Health [Denmark]	26,687 men<br>29,308 women	50-64	17.5	192-item FFQ	Whole-grain bread<br>Rye bread	Age; education; smoking; pack years; physical activity; intakes of alcohol, fruit, vegetables, red meat, processed meat; body mass index	Pancreatic cancer incidence [446 cases]<br><br>Hazard Ratio for dose-response: "per serving" (50g for rye bread; 40g for whole-grain bread)
Skeie 2016	Scandinavian HELGA cohort [Norway, Denmark, Sweden]	113,993 men (33.8%) and women (66.2%)	30-64	11	Norway: 88-item FFQ<br>Sweden: 98-item FFQ<br>Denmark: 173-item FFQ	Whole-grain bread	Age; sex; smoking; carbohydrate and non-carbohydrate energy intake; intakes of cakes and biscuits, alcohol, processed meat, and different whole-grain products	Esophageal cancer incidence [112 cases]<br><br>Hazard Ratio: Tertile 3 vs. Tertile 1; Dose-response: per 25g/day higher intake
Sonestedt 2008	Malmo Diet and Cancer cohort	15,773 women	46-75	10.3	168-item FFQ	Low-fiber bread<br>High-fiber bread	Age; smoking; season; diet interviewer; method version; total energy intake; body weight; education; leisure-time physical activity; household activity; alcohol consumption; age at	Breast cancer incidence [544 cases]<br><br>Hazard Ratio: Quintile 5 vs. Quintile 1

							menopause; parity; hormone replacement therapy	
Torfadottir 2012	Age Gene Environment Susceptibility (AGES)-Reykjavik Cohort Study	1,980 men	33-79	Follow-up from 2002-2006 through 2009	FFQ	Rye bread<br>Whole wheat bread	Age; education; family history of prostate disease; going to a physician regularly; height in midlife; body mass index in midlife; type 2 diabetes in midlife; intakes of fish, fish liver oil, meat, milk	Prostate cancer incidence [133 total cases; 27 advanced cases]<br><br>Hazard Ratio: “Daily or more” vs. “less than daily”
Von Ruesten 2013	European Prospective Investigation into Cancer and Nutrition (EPIC)-Potsdam [Germany]	9,098 men<br>14,433 women	35-65	8	148-item FFQ	Whole-grain bread (including dark or whole-grain rolls)<br>Other bread (rye/brown bread, white or wheat toast, white rolls, croissant/crispbread/rye pretzel)	Age; sex; smoking; leisure-time physical activity; body mass index; waist-to-hip ratio; education; vitamin supplementation; non-consumption of the respective food group; total energy intake	Total cancer incidence<br><br>Hazard Ratio:<br>Dose-response:<br>Per 50g/day increase

BMI = body mass index; FFQ = Food Frequency Questionnaire

Supplemental Table 2. Quality of Study Assessment – Newcastle-Ottawa Scale

Publication	Selection				Comparability	Outcome			Total score
	Representativeness of the exposed cohort	Selection of the non-exposed cohort	Ascertainment of exposure	Demonstration that outcome of interest was not present at start of study	Comparability of cohorts on the basis of design or analysis	Assessment of outcome	Was follow-up long enough for outcome to occur	Adequacy of follow-up of cohorts	
Studies included in systematic review and primary meta-analysis									
Abe 2014	✷	✷	✷	✷	✷✷	✷	✷	✷	9
Bakken 2016	-	✷	✷	✷	✷✷	✷	✷	-	7
Cordova 2023	✷	✷	✷	✷	✷✷	✷	✷	-	8
Haraldsdottir 2018	✷	✷	✷	✷		✷	✷	-	6
Jacobs 2001	✷	✷	✷	-	✷✷	✷	✷	-	7
Jin 2023	✷	✷	✷	✷	✷✷	✷	✷	✷	9
Lan 2021	✷	✷	✷	✷	✷✷	✷	✷	-	8
Skeie 2016	✷	✷	✷	-	✷	✷	✷	-	6
Sonestedt 2008	✷	✷	✷	✷	✷✷	✷	✷	-	8
Torfadottir 2012	✷	✷	✷	-	-	✷	-	-	4
Studies included in systematic review and supplemental meta-analysis									
Appleby 2002	-	✷	-	✷	✷	✷	✷	-	5
Egeberg 2011	✷	✷	✷	✷	✷✷	✷	✷	✷	9
Hedelin 2011	✷	✷	✷	✷	✷	✷	✷	-	7
Johnsen 2015	✷	✷	✷	✷	✷	✷	✷	-	7
Kyro 2013	✷	✷	✷	-	✷✷	✷	✷	-	7
Larsson 2005	✷	✷	✷	✷	✷	✷	✷	-	7
Sanjoaquin 2004	-	✷	-	✷	✷	✷	✷	✷	6
Studies included in systematic review but not included in meta-analysis (Dose-response data only)									
Aarestrup 2012	-	✷	✷	✷	✷	✷	✷	✷	7

Anderson 2020	-	✷	✷	✷	✷	✷	✷	✷	7
Egeberg 2010	✷	✷	✷	✷	✷	✷	✷	-	7
Farvid 2016	✷	✷	✷	✷	✷✷	✷	✷	✷	9
Papadimitriou 2022	✷	✷	✷	✷	✷✷	✷	✷	-	8
Schacht 2021	✷	✷	✷	✷	✷✷	✷	✷	-	8
Von Ruesten 2013	✷	✷	✷	✷	✷✷	✷	-	-	7

Selection:
1) Representativeness of exposed cohort
Star assigned if cohort was truly or somewhat representative of the community/population from which it was drawn.
2) Selection of non-exposed cohort
Star assigned where non-exposed persons were drawn from the same population as the exposed participants.
3) Ascertainment of exposure
Star assigned where diets were assessed using structured interviews or validated diet records (such as a Food Frequency Questionnaire).
4) Demonstration that outcome was not present at start of study:
Star assigned where participants with cancer at the time the study started were excluded.

Comparability:
1) Comparability of cohorts on the basis of the design or analysis
One star assigned where both age and smoking were controlled for in analyses.

Second star assigned where control for at least three of the following were controlled for in the analyses: physical activity; body mass index; fiber; meat, glycemic index/load; fruits/vegetables; total energy intake.

Outcome:
1) Assessment of outcome
Star assigned where outcomes were identified through medical records/record linkage.
2) Was follow-up long enough for outcomes to occur
Star assigned where mean years of follow-up was >10 years.
3) Adequacy of follow up of cohorts
Star assigned where the follow-up rate was >80%. Stars were not assigned where these data were not reported or could not be ascertained from outcome data provided.

PRISMA 2020 for Abstracts Checklist

Section and Topic	Item #	Checklist item	Reported (Yes/No)
**TITLE**			
Title	1	Identify the report as a systematic review.	Yes
**BACKGROUND**			
Objectives	2	Provide an explicit statement of the main objective(s) or question(s) the review addresses.	Yes
**METHODS**			
Eligibility criteria	3	Specify the inclusion and exclusion criteria for the review.	Yes
Information sources	4	Specify the information sources (e.g. databases, registers) used to identify studies and the date when each was last searched.	Yes
Risk of bias	5	Specify the methods used to assess risk of bias in the included studies.	Yes
Synthesis of results	6	Specify the methods used to present and synthesise results.	Yes
**RESULTS**			
Included studies	7	Give the total number of included studies and participants and summarise relevant characteristics of studies.	Yes
Synthesis of results	8	Present results for main outcomes, preferably indicating the number of included studies and participants for each. If meta-analysis was done, report the summary estimate and confidence/credible interval. If comparing groups, indicate the direction of the effect (i.e. which group is favoured).	Yes
**DISCUSSION**			
Limitations of evidence	9	Provide a brief summary of the limitations of the evidence included in the review (e.g. study risk of bias, inconsistency and imprecision).	No
Interpretation	10	Provide a general interpretation of the results and important implications.	Yes
**OTHER**			
Funding	11	Specify the primary source of funding for the review.	No
Registration	12	Provide the register name and registration number.	Yes

PRISMA 2020 Checklist

	Item #	Checklist item	Location where item is reported
**TITLE**			
Title	1	Identify the report as a systematic review.	Page 1
**ABSTRACT**			
Abstract	2	See the PRISMA 2020 for Abstracts checklist.	Pages 3-4
**INTRODUCTION**			
Rationale	3	Describe the rationale for the review in the context of existing knowledge.	Pages 5-6
Objectives	4	Provide an explicit statement of the objective(s) or question(s) the review addresses.	Page 6
**METHODS**			
Eligibility criteria	5	Specify the inclusion and exclusion criteria for the review and how studies were grouped for the syntheses.	Page 7
Information sources	6	Specify all databases, registers, websites, organisations, reference lists and other sources searched or consulted to identify studies. Specify the date when each source was last searched or consulted.	Page 7
Search strategy	7	Present the full search strategies for all databases, registers and websites, including any filters and limits used.	Page 7
Selection process	8	Specify the methods used to decide whether a study met the inclusion criteria of the review, including how many reviewers screened each record and each report retrieved, whether they worked independently, and if applicable, details of automation tools used in the process.	Page 7-8
Data collection process	9	Specify the methods used to collect data from reports, including how many reviewers collected data from each report, whether they worked independently, any processes for obtaining or confirming data from study investigators, and if applicable, details of automation tools used in the process.	Pages 7-8
Data items	10a	List and define all outcomes for which data were sought. Specify whether all results that were compatible with each outcome domain in each study were sought (e.g. for all measures, time points, analyses), and if not, the methods used to decide which results to collect.	Page 7
	10b	List and define all other variables for which data were sought (e.g. participant and intervention characteristics, funding sources). Describe any assumptions made about any missing or unclear information.	Pages 7-8
Study risk of bias assessment	11	Specify the methods used to assess risk of bias in the included studies, including details of the tool(s) used, how many reviewers assessed each study and whether they worked independently, and if applicable, details of automation tools used in the process.	Page 9
Effect measures	12	Specify for each outcome the effect measure(s) (e.g. risk ratio, mean difference) used in the synthesis or presentation of results.	Pages 8-9
Synthesis methods	13a	Describe the processes used to decide which studies were eligible for each synthesis (e.g. tabulating the study intervention characteristics and comparing against the planned groups for each synthesis (item #5)).	Page 7
	13b	Describe any methods required to prepare the data for presentation or synthesis, such as handling of missing summary statistics, or data conversions.	Page 7
	13c	Describe any methods used to tabulate or visually display results of individual studies and syntheses.	Pages 8-9
	13d	Describe any methods used to synthesize results and provide a rationale for the choice(s). If meta-analysis was performed, describe the model(s), method(s) to identify the presence and extent of statistical heterogeneity, and software package(s) used.	Pages 8-9
	13e	Describe any methods used to explore possible causes of heterogeneity among study results (e.g. subgroup analysis, meta-regression).	Page 9
	13f	Describe any sensitivity analyses conducted to assess robustness of the synthesized results.	Page 9
Reporting bias assessment	14	Describe any methods used to assess risk of bias due to missing results in a synthesis (arising from reporting biases).	N/A
Certainty assessment	15	Describe any methods used to assess certainty (or confidence) in the body of evidence for an outcome.	Page 9

PRISMA 2020 Checklist

	Item #	Checklist item	Location where item is reported
**RESULTS**			
Study selection	16a	Describe the results of the search and selection process, from the number of records identified in the search to the number of studies included in the review, ideally using a flow diagram.	Pages 9-10
	16b	Cite studies that might appear to meet the inclusion criteria, but which were excluded, and explain why they were excluded.	Pages 9-10
Study characteristics	17	Cite each included study and present its characteristics.	Table 1<br>Suppl Table 1
Risk of bias in studies	18	Present assessments of risk of bias for each included study.	Pages 16-20
Results of individual studies	19	For all outcomes, present, for each study: (a) summary statistics for each group (where appropriate) and (b) an effect estimate and its precision (e.g. confidence/credible interval), ideally using structured tables or plots.	Tables 1 and 2
Results of syntheses	20a	For each synthesis, briefly summarise the characteristics and risk of bias among contributing studies.	Pages 16-20
	20b	Present results of all statistical syntheses conducted. If meta-analysis was done, present for each the summary estimate and its precision (e.g. confidence/credible interval) and measures of statistical heterogeneity. If comparing groups, describe the direction of the effect.	Figures 1 and 2<br>Suppl Figures 1 and 3
	20c	Present results of all investigations of possible causes of heterogeneity among study results.	
	20d	Present results of all sensitivity analyses conducted to assess the robustness of the synthesized results.	Page 20
Reporting biases	21	Present assessments of risk of bias due to missing results (arising from reporting biases) for each synthesis assessed.	N/A
Certainty of evidence	22	Present assessments of certainty (or confidence) in the body of evidence for each outcome assessed.	Pages 24-26
**DISCUSSION**			
Discussion	23a	Provide a general interpretation of the results in the context of other evidence.	Pages 20-26
	23b	Discuss any limitations of the evidence included in the review.	Pages 24-25
	23c	Discuss any limitations of the review processes used.	Pages 24-25
	23d	Discuss implications of the results for practice, policy, and future research.	Page 23
**OTHER INFORMATION**			
Registration and protocol	24a	Provide registration information for the review, including register name and registration number, or state that the review was not registered.	Pages 4 and 6
	24b	Indicate where the review protocol can be accessed, or state that a protocol was not prepared.	Pages 4 and 6
	24c	Describe and explain any amendments to information provided at registration or in the protocol.	N/A

PRISMA 2020 Checklist

	Item #	Checklist item	Location where item is reported
Support	25	Describe sources of financial or non-financial support for the review, and the role of the funders or sponsors in the review.	Page 2
Competing interests	26	Declare any competing interests of review authors.	Page 26
Availability of data, code and other materials	27	Report which of the following are publicly available and where they can be found: template data collection forms; data extracted from included studies; data used for all analyses; analytic code; any other materials used in the review.	Pages 26-27

From: Page MJ, McKenzie JE, Bossuyt PM, Boutron I, Hoffmann TC, Mulrow CD, et al. The PRISMA 2020 statement: an updated guideline for reporting systematic reviews. BMJ 2021;372:n71. doi: 10.1136/bmj.n71

For more information, visit: http://www.prisma-statement.org/
